# Bibliometric and content analysis of the Cochrane Complementary Medicine Field specialized register of controlled trials

**DOI:** 10.1186/2046-4053-2-51

**Published:** 2013-07-04

**Authors:** L Susan Wieland, Eric Manheimer, Margaret Sampson, Jabez Paul Barnabas, Lex M Bouter, Kiho Cho, Myeong Soo Lee, Xun Li, Jianping Liu, David Moher, Tetsuro Okabe, Elizabeth D Pienaar, Byung-Cheul Shin, Prathap Tharyan, Kiichiro Tsutani, Daniëlle A van der Windt, Brian M Berman

**Affiliations:** 1 University of Maryland School of Medicine, Baltimore, MD, USA; 2 Children’s Hospital of Eastern Ontario, Ottawa, Canada; 3 Christian Medical College, Vellore, India; 4 VU University Amsterdam, Amsterdam, The Netherlands; 5 Kyunghee University, Seoul, South Korea; 6 Korea Institute of Oriental Medicine, Seoul, South Korea; 7 Beijing University of Chinese Medicine, Beijing, China; 8 Ottawa Hospital Research Institute, University of Ottawa, Ottawa, Canada; 9 University of Tokyo, Tokyo, Japan; 10 South African Cochrane Centre, South African Medical Research Council, Cape Town, South Africa; 11 Pusan National University, Busan, South Korea; 12 Keele University, Staffordshire ST5 5BG, UK

**Keywords:** Complementary medicine, Randomized controlled trials, Data collection, Registries

## Abstract

**Background:**

The identification of eligible controlled trials for systematic reviews of complementary and alternative medicine (CAM) interventions can be difficult. To increase access to these difficult to locate trials, the Cochrane Collaboration Complementary Medicine Field (CAM Field) has established a specialized register of citations of CAM controlled trials. The objective of this study is to describe the sources and characteristics of citations included in the CAM Field specialized register.

**Methods:**

Between 2006 and 2011, regular searches for citations of CAM trials in MEDLINE and the Cochrane Central Register of Controlled Trials (CENTRAL) were supplemented with contributions of controlled trial citations from international collaborators. The specialized register was ‘frozen’ for analysis in 2011, and frequencies were calculated for publication date, language, journal, presence in MEDLINE, type of intervention, and type of medical condition.

**Results:**

The CAM Field specialized register increased in size from under 5,000 controlled trial citations in 2006 to 44,840 citations in 2011. Most citations (60%) were from 2000 or later, and the majority (71%) were reported in English; the next most common language was Chinese (23%). The journals with the greatest number of citations were CAM journals published in Chinese and non-CAM nutrition journals published in English. More than one-third of register citations (36%) were not indexed in MEDLINE. The most common CAM intervention type in the register was non-vitamin, non-mineral dietary supplements (e.g., glucosamine, fish oil) (34%), followed by Chinese herbal medicines (e.g., *Astragalus membranaceus*, *Schisandra chinensis*) (27%).

**Conclusions:**

The availability of the CAM Field specialized register presents both opportunities and challenges for CAM systematic reviewers. While the register provides access to thousands of difficult to locate trial citations, many of these trials are of low quality and may overestimate treatment effects. When including these trials in systematic reviews, adequate analysis of their risk of bias is of utmost importance.

## Background

Complete identification of eligible controlled trials is an essential step in conducting a systematic review, and finding and collecting citations of controlled trials have been aims of the Cochrane Collaboration from its inception 20 years ago. As part of this mission, the Collaboration developed the Cochrane Central Register of Controlled Trials (CENTRAL), a searchable database of citations of controlled trials [1]. The Collaboration has agreements with the publishers of MEDLINE and EMBASE, ensuring that all citations of controlled trials from those databases are republished in CENTRAL. Cochrane entities (e.g., Cochrane Review Groups) also regularly submit citations of controlled trials to CENTRAL, ensuring that CENTRAL contains trial citations from multiple sources, including not only MEDLINE and EMBASE, but also regional and subject-specific databases, as well as trial citations not included in databases. Because of the Collaboration’s extensive efforts at identification of trials from a range of sources, CENTRAL is considered to be the most comprehensive source of citations of controlled trials for inclusion in systematic reviews [1].

The complete identification of eligible controlled trials can be particularly challenging for systematic reviews of CAM interventions. The disadvantage of relying exclusively upon sources such as MEDLINE for trial identification is illustrated by research conducted by Egger and colleagues. Egger and colleagues [2] analyzed the characteristics of 1,635 controlled trials included in a group of *n* = 159 systematic reviews, including both conventional and CAM-related reviews. They found that in CAM-related systematic reviews, the proportion of non-MEDLINE-indexed trials (41%) was approximately twice that proportion seen in conventional medicine systematic reviews (21%). If the systematic review authors had searched only MEDLINE for trials, they would have missed many trials, possibly including some important trials, and the proportion missing from CAM reviews would have been double the proportion missing from conventional medicine reviews. Earlier research using various ‘gold standard sets’ of known trials for specific CAM interventions found that the percentage of known trials included in MEDLINE was 58% for acupuncture trials [3], 31% for ginkgo trials [4], and 17% for homeopathy trials [4]. Ensuring that CENTRAL contains both MEDLINE and non-MEDLINE citations of CAM-related controlled trials is therefore important for the unbiased conduct of Cochrane systematic reviews of CAM interventions.

The CAM Field maintains a specialized register of citations of controlled trials of CAM interventions, which is a ‘subregister’ of CENTRAL. In 1998, a bibliometric analysis of the CAM Field specialized register described it as containing 3,774 controlled trials [5]. In 2006, the CAM Field began an active program to improve the scope and size of the CAM Field specialized register. CAM Field staff and international partners in this endeavor performed searches of bibliographic databases and paper journals, and CAM Field staff performed extensive quality checks and de-duplication of all identified citations. As a result of these efforts, the CAM Field register contained a total of 43,310 CAM Field specialized register citations as of Issue 1, 2012, of *The Cochrane Library*. CAM Field specialized register citations can be retrieved from the Cochrane Central Register of Controlled Trials (CENTRAL) database of *The Cochrane Library* by searching for the tag ‘SR-COMPMED’ in all text. The objective of this study is to describe the sources and characteristics of the trial citations included in the CAM Field specialized register.

## Methods

In 2011 we drew up a detailed protocol (see Additional file 1) that described the eligibility criteria for including citations and the methods we had used to build the register. The protocol also pre-specified the methods we planned to use in examining the characteristics of the citations in the register. A summary of these methods, as well as the details of contributions of trial citations by international collaborators, is presented below.

### Eligibility criteria

All citations in the CAM Field specialized register are required to meet the following two inclusion criteria: (1) they must be reports of controlled trials, and (2) they must be CAM-related.

We considered controlled trials to be studies meeting the inclusion criteria for CENTRAL that were formulated and agreed upon in November 1992 and are published in Chapter 6.3 of the Cochrane Handbook [6]. We considered trials to be CAM-related if they described interventions that are outside the practices and theories of disease and healing that are intrinsic to the conventional Western medical model [7]. To retrieve citations of CAM trials from MEDLINE and CENTRAL, we relied upon the CAM on PubMed search strategy, which was jointly developed by the US National Library of Medicine and the US National Institutes of Health, National Center for Complementary and Alternative Medicine, and introduced in PubMed in 2001 [8,9]. In cases where some uses of the intervention are accepted within conventional Western medicine and others are not (e.g., vitamin supplementation), the CAM on PubMed search strategy generally does not distinguish between conventional and unconventional uses of an intervention. Therefore, for classifying interventions as conventional or CAM, we followed the same operational criteria we had previously developed for classifying Cochrane systematic reviews as conventional or CAM [10]. Some of the major decisions about the scope of CAM were as follows: we excluded vitamins and other supplements that are administered parenterally in hospital settings, we excluded dietary supplementation for treatment or prevention of medically diagnosed deficiency states (e.g., iron supplementation for preventing or treating iron deficiency), and we excluded vitamin supplements for preventing or treating disease in countries where vitamin deficiency is widespread (e.g., vitamin A for treating measles in children in Niger). We included vitamins for other conditions, even vitamins that are accepted for the prevention or treatment of specific disorders (e.g., folic acid for preventing neural tube defects). In general, we decided that we should be over-inclusive rather than under-inclusive with vitamin therapies, aside from the three major exclusions detailed above, and therefore some of the vitamin trials in the database would not be accepted as CAM by most people. Finally, we excluded exercise interventions with the exception of mind-body exercise (e.g., yoga), and we excluded conventional psychotherapies. A full description of the CAM Field operational definition of CAM has been published previously (see Additional file 2) [10].

### Methods for building the register of trials

We began the expansion of the CAM Field specialized register of trials by building upon the reference management database of nearly 5,000 CAM controlled trial citations developed during the 1990s and early 2000s by Vickers and colleagues [5]. In 2006, we began regular searches of MEDLINE in PubMed using the CAM on PubMed search strategy. In 2008, an information specialist translated the CAM on PubMed search strategy into a format for use in CENTRAL, and we replaced searches of PubMed with regular searches of CENTRAL. The rationale for replacing searches of PubMed with searches of CENTRAL is that CENTRAL includes not only controlled trial citations from MEDLINE, but also controlled trial citations from multiple other sources. These other citations, which may be from other databases or from difficult to locate sources such as trial proceedings, are identified by Cochrane contributors around the world and contributed to CENTRAL. All Cochrane groups then search CENTRAL in order to identify relevant citations that others have contributed. We began by searching CENTRAL from inception and then searched newly added citations in each subsequent issue of CENTRAL.

An important subset of CAM is traditional medicine, defined by the World Health Organization as “the sum total of knowledge, skills and practices based on the theories, beliefs and experiences indigenous to different cultures that are used to maintain health, as well as to prevent, diagnose, improve or treat physical and mental illnesses. Traditional medicine that has been adopted by other populations (outside its indigenous culture) is often termed alternative or complementary medicine” [11]. Different countries often have their own forms of traditional medicine (e.g., traditional Chinese medicine, traditional Korean medicine). Because we did not have access to the traditional medicine trial reports that are published in regional or national databases and journals, in 2008 we began contacting Cochrane colleagues and contributors to solicit the contributions of citations of traditional medicine trials published in their regions. These efforts are described below. Searches of PubMed, and then of CENTRAL, were thus complemented with searches of bibliographic databases and journals conducted by several international groups who contributed citations of trial reports to the CAM Field for inclusion in the CAM Field register. Citations provided by contributing organizations were not restricted by publication year. As described below, two of these contributing groups also provided PDFs of the full text publications for all identified citations. Collaborators are listed below in order of numbers of citations submitted to the CAM Field for the specialized register.

### Traditional Chinese medicine (TCM) trials identified by Chinese collaborators

Beginning in 2008, staff at the Center for Evidence-Based Medicine of the Beijing University of Chinese Medicine, under the direction of Jianping Liu, searched both electronic databases and Chinese journals to identify citations of controlled trials of TCM interventions. The journal titles, article titles, and abstracts (if available) of all identified citations were translated into English, entered into a reference management database with added topic keywords, and submitted to the CAM Field for inclusion in the specialized register. The full text report was also submitted to the CAM Field for each citation in a PDF format.

### Trials from CAM-specific databases identified by Canadian collaborators

In 2008, information specialists under the direction of David Moher of the Ottawa Hospital Research Institute undertook a project to search several specialized databases for difficult to locate controlled trials of CAM interventions, and these searches were replicated in 2010 [12]. All identified citations were imported into a reference management database, information about the source database and the type of CAM intervention was included for each citation, and the database was submitted to the CAM Field for inclusion in the CAM Field specialized register.

### Traditional medicine trials identified by Korean collaborators

In 2010, researchers at the Korea Institute of Oriental Medicine, under the direction of Myeong Soo Lee, searched both electronic databases and journals to identify citations of controlled trials of traditional medicine interventions conducted in Korea and primarily published in non-MEDLINE journals. Initial searches were focused on identifying trials of acupuncture [13] and ginseng, and were then expanded to include all other CAM interventions. The journal titles, article titles, and abstracts (if available) of all identified citations were translated into English and entered into a reference management database with topic keywords, and the citations were submitted to the CAM Field. For each citation, the full text publication was also submitted to the CAM Field in a PDF format.

### Kampo trials identified by Japanese collaborators

Kampo is the Japanese adaptation of traditional Chinese medicine. While Kampo uses most of the interventions of Chinese medicine, including acupuncture and moxibustion, its primary focus is on the study and evaluation of traditional herbal medicines. In 2001, the Japan Society for Oriental Medicine undertook a project to collect controlled trial evidence on Kampo interventions through searches of both electronic databases and journals [14]. As randomized controlled trials of Kampo interventions are identified, structured abstracts are prepared for each trial, and the citations and structured abstracts are published online in English. In March 2011, one of the leaders of this initiative, Kiichiro Tsutani of the University of Tokyo, provided permission for the CAM Field staff to incorporate the citations associated with these Kampo trials into the CAM Field specialized register together with links to the online structured abstracts.

### Ayurveda and other CAM-related trials identified by Indian collaborators

The South Asian Database of Controlled Clinical Trials (SADCCT) is an online database of citations of controlled trials that have been conducted in countries for which the South Asian Cochrane Network & Centre is the reference Cochrane Centre, including Afghanistan, Bangladesh, Bhutan, India, the Maldives, Nepal, Pakistan, and Sri Lanka. The SADCCT was developed by searching South Asian journals and conference proceedings for all controlled trials [15]. In 2011, staff at the South Asian Cochrane Network & Centre, under the direction of Prathap Tharyan, identified and forwarded citations of South Asian trials of Ayurveda, Unani, Siddha, and other CAM interventions included in the SADCCT, and in 2012 staff at the CAM Field identified additional trial citations from the online SADCCT.

### CAM trials identified by African collaborators

The African Trials Register is a database of citations of controlled trials conducted in Africa. It has been developed at the South African Cochrane Centre by the Cochrane Centre staff searching African journals and electronic bibliographic databases [16,17]. In 2011, citations of CAM-related trials included in the African Trials Register were identified by Elizabeth Pienaar, information specialist at the South African Cochrane Centre, and a database of citations was forwarded to the CAM Field.

### Methods for examining the contents of the register of trials

In August 2011, we suspended additions of new search results to the CAM Field specialized register and began an intensive program of cleaning and updating the register in preparation for analysis, focusing on detection and removal of any non-CAM or non-controlled trial citations, deduplication of register citations, identification of whether each register citation was present or absent in MEDLINE, and standardization of journal names (see Additional file 1 for details of procedures used). Because EMBASE is a second major database from which controlled trials are automatically downloaded to CENTRAL, we also wished to characterize the EMBASE coverage of CAM Field register citations. However, register citations do not contain EMBASE identifiers, and we did not have the resources to comprehensively check all citations for presence in EMBASE. We therefore estimated the proportion of register citations present in EMBASE by taking a random sample of 200 register records and searching EMBASE for each citation in the sample. We used the same random sample to estimate the overlap in coverage between MEDLINE and EMBASE, and the proportion of register citations not present in either MEDLINE or EMBASE.

We sorted journal titles by frequency and the 100 journals associated with the greatest number of trial citations were classified as either CAM or conventional in focus, using the classification method described in the protocol (see Additional file 1). For each of the 25 CAM and 25 conventional journals with the greatest number of trial citations, we determined the language of publication and whether the journal was indexed in MEDLINE. In addition to characterizing the journals with the greatest number of citations, we also examined the distribution of citations across all journals in the register to determine to what extent citations are scattered across journals overall.

To characterize register citations by CAM intervention, we chose 21 different types of CAM interventions within five broad categories. We based the 5 broad categories and the 21 intervention types within categories upon the CAM Field topics list for Cochrane reviews of CAM interventions [10] as well as other classifications of CAM interventions (e.g., the classifications of CAM interventions used in the 2007 NHIS survey of use of CAM in the United States [18]). We then developed subject searches for each of the 21 types of CAM interventions by parsing the 2006 translation of the CAM on PubMed search strategy into the CAM intervention topic areas. We also consulted additional sources to identify any supplementary terms and to help understand and delineate between the CAM intervention topic areas. Searches were run and tested in MEDLINE, using each relevant term. An information specialist developed the MEDLINE version of each search strategy, which was then peer reviewed by an independent information specialist using the PRESS standard [19]. Searches were then adapted so that they could be used to search the reference management database containing the register. MEDLINE Medical Subject Heading (MeSH) terms and free text terms were sought in all database fields. The search strategies for identifying each of the 21 different types of CAM interventions are included as an additional file so that these strategies will be publicly available to CAM systematic reviewers developing searches for any of these classes of therapies (see Additional file 3).

To characterize register citations by medical conditions, we used the 25 categories of medical conditions listed in the browse list on the home page of *The Cochrane Library*[20]. We developed subject searches for each medical condition category by consulting Cochrane reviews grouped under each of these 25 categories to ascertain relevant search terms and concepts, and additional sources to identify any supplementary terms. Searches were run and tested in MEDLINE in the same manner as described above for the CAM intervention searches, although these searches on medical conditions were not peer reviewed. Search strategies were then adapted so that they could be used to search the CAM register and augmented with additional free text synonyms for relevant medical conditions. Both MeSH and free text terms were sought in all reference management database fields.

## Results

At the time of our analysis, the CAM Field register of trials included 44,840 citations of CAM trials, which represented approximately 6% of the total number of all trial citations in CENTRAL [21]. Of these 44,840 CAM trial citations, 15,990 (36%) are not included in MEDLINE. Among the random sample of 200 register citations checked for EMBASE status, 63/200 (31.5%; 95% CI 25% to 38%) are not included in EMBASE. Among these 63 citations not included in EMBASE, only 7/63 (11.1%; 95% CI 3% to 19%) are included in MEDLINE. Among the 69/200 sample citations not included in MEDLINE, only 13/69 (18.8%; 95% CI 10% to 28%) are included in EMBASE. Overall, 56/200 (28%; 95% CI 22% to 34%) of sample citations are not included in either MEDLINE or EMBASE.

**Figure 1:**
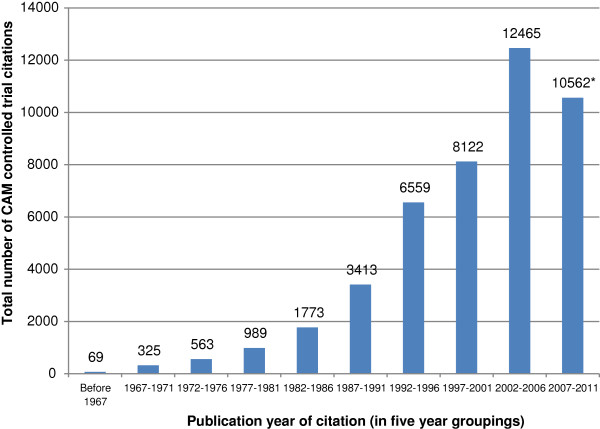
**Number of citations in the CAM Field specialized register by citation publication year.** * The drop in the number of trial citations for 2007–2011 may be partially due to incomplete indexing of trials published in this recent time period and/or may be partially due to our having suspended additions of new search results to the CAM Field specialized register in August 2011.

There is a substantial increase in the numbers of trial citations published for each 5-year time period, and the majority of the citations are from more recent publication years (see Figure 1). This increase in the numbers of trial citations included in the CAM Field register over publication year time periods corresponds to a similar increase over publication year periods seen overall in CENTRAL [22].

The most common languages in the register after English are Chinese (*n* = 10,376; 23%), German (*n* = 963; 2%), Korean (*n* = 330; 0.7%), Japanese (*n* = 312; 0.7%), and Russian (*n* = 227; 0.5%). The representation of citations in languages other than English probably reflects both the number of trials published in that language as well as our methods of sourcing the trial citations for the register. For example, the number of trials in Chinese reflects both the fact that Chinese journals publish a large number of trials of traditional Chinese medicine interventions (e.g., acupuncture, Chinese herbal medicine) [23] and also the fact that we have collaborated with our partner institution in China, since 2008, to search Chinese journals and databases to identify these trials. More recent collaborations have resulted in the identification and inclusion in the register of traditional medicine trial citations from other countries (see Table 1). Searches of trials by several of these collaborating institutions are ongoing.

**Table 1:** Source of citations in the CAM Field specialized register

**Source of citations**	**All citations *n *(% of citations in register)**	**Non-MEDLINE N (% of non-MEDLINE citations in register)**
Contributing organization		
Beijing University of Chinese Medicine	6,484 (14%)	6,183 (39%)
Ottawa Hospital Research Institute	2,967 (7%)	2,777 (17%)
Japan Society for Oriental Medicine	351 (1%)	293 (2%)
Korea Institute of Oriental Medicine	307 (1%)	304 (2%)
South Asian Cochrane Centre & Network	71 (<1%)	31 (<1%)
South African Cochrane Centre	26 (<1%)	0 (0)
**Total citations from contributing organizations**	**10,206 (23%)**	**9,588 (60%)**
**Total citations from other sources (e.g., searches of CENTRAL)**	**34,634 (77%)**	**6,402 (40%)**
**Totals**	**44,840 (100%)**	**15,990 (100%)**

The 25 conventional journal titles and 25 CAM journal titles with the largest number of citations in the register are listed in Tables 2 and 3, respectively. Nearly all (24/25) of the top conventional medicine journals are MEDLINE-indexed, while only 12/25 of the top 25 CAM journals are MEDLINE-indexed. Together, these 50 journals account for 13,731 trials (30.6% of the total) and 8,445 of these trial citations (61.5%) are MEDLINE-indexed. The clinical focus of the conventional journals was concentrated in nutrition (*n* = 8), general and internal medicine (*n* = 4), and pediatrics (*n* = 3). The clinical focus of the top 25 CAM journals was concentrated in TCM (*n* = 20).

**Table 2:** Twenty-five conventional medicine journals with the most citations in the CAM Field specialized register

Journal name*	**Number of citations (% of citations in register)**	**Language of full text**	**MEDLINE-indexed**
The American Journal of Clinical Nutrition	1,027 (2.3)	English	Yes
European Journal of Clinical Nutrition	381 (0.8)	English	Yes
The Journal of Nutrition	362 (0.8)	English	Yes
The British Journal of Nutrition	310 (0.7)	English	Yes
Lancet	247 (0.6)	English	Yes
BMJ	223 (0.5)	English	Yes
Pain	182 (0.4)	English	Yes
Journal of Consulting and Clinical Psychology	178 (0.4)	English	Yes
Behaviour Research and Therapy	170 (0.4)	English	Yes
Journal of Clinical Rehabilitative Tissue Engineering Research (Zhong Guo Zu Zhi Gong Cheng Yan Jiu Yu Lin Chuang Kang Fu)	166 (0.4)	Chinese	No
Archives of Physical Medicine and Rehabilitation	164 (0.4)	English	Yes
Journal of the American College of Nutrition	156 (0.3)	English	Yes
Diabetes Care	144 (0.3)	English	Yes
JAMA: Journal of the American Medical Association	136 (0.3)	English	Yes
Atherosclerosis	132 (0.3)	English	Yes
Journal of Clinical Endocrinology and Metabolism	131 (0.3)	English	Yes
Nutrition (Burbank, Los Angeles County, Calif.)	128 (0.3)	English	Yes
Pediatrics	127 (0.3)	English	Yes
The Journal of Pediatrics	121 (0.3)	English	Yes
Lipids	119 (0.3)	English	Yes
The New England Journal of Medicine	111 (0.2)	English	Yes
Arzneimittel-Forschung	110 (0.2)	English, German†	Yes
Medicine and Science in Sports and Exercise	91 (0.2)	English	Yes
Journal of Pediatric Gastroenterology and Nutrition	90 (0.2)	English	Yes
Journal of the American Dietetic Association	88 (0.2)	English	Yes
Total	5,094 (11.4)		

*Totals include named journals and any predecessor journals that were continued by the named journal.

†Articles are published in either German or English, and abstracts are available in both languages.

**Table 3:** Twenty-five complementary medicine journals with the most citations in the CAM Field specialized register

Journal name*	**Number of citations (% of citations in register)**	**Language of full text**	**MEDLINE-indexed**
Chinese Journal of Integrated Traditional and Western Medicine (Zhong Guo Zhong Xi Yi Jie He Za Zhi)	1,809 (4.0)	Chinese	Yes
Chinese Journal of Information on Traditional Chinese Medicine (Zhong Guo Zhong Yi Yao Xin Xi Za Zhi)	1,049 (2.3)	Chinese	No
Chinese Acupuncture & Moxibustion (Zhongguo zhen jiu)	1,021 (2.3)	Chinese	No
Shanghai Journal of Acupuncture and Moxibustion (Shang Hai Zhen Jiu Za Zhi)	557 (1.2)	Chinese	No
Journal of Traditional Chinese Medicine	367 (0.8)	English	Yes
Journal of Alternative and Complementary Medicine	357 (0.8)	English	Yes
Chinese Journal of Integrated Traditional and Western Medicine on Liver Diseases (Zhong Xi Yi Jie He Gan Bing Za Zhi)	356 (0.8)	Chinese	No
Modern Journal of Integrated Traditional Chinese and Western Medicine (Xian Dai Zhong Xi Yi Jie He Za Zhi)	350 (0.8)	Chinese	No
Journal of Manipulative and Physiological Therapeutics	274 (0.6)	English	Yes
Chinese Traditional Patent Medicine (Zhong Cheng Yao)	231 (0.5)	Chinese	No
China Journal of Chinese Materia Medica (Zhong Guo Zhong Yao Za Zhi)	189 (0.4)	Chinese	Yes
Shanghai Journal of Traditional Chinese Medicine (Shang Hai Zhong Yi Yao Za Zhi)	186 (0.4)	Chinese	No
Hebei Journal of Traditional Chinese Medicine (He Bei Zhong Yi)	184 (0.4)	Chinese	No
Chinese Journal of Integrative Medicine	178 (0.4)	English	Yes
The American Journal of Chinese Medicine	169 (0.4)	English	Yes
Shandong Journal of Traditional Chinese Medicine (Shan Dong Zhong Yi Za Zhi)	163 (0.4)	Chinese	No
Complementary Therapies in Medicine	150 (0.3)	English	Yes
World Journal of Acupuncture-Moxibustion	147 (0.3)	English	No
China Journal of Traditional Chinese Medicine and Pharmacy (Zhong Hua Zhong Yi Yao Za Zhi)	146 (0.3)	Chinese	No
Jiangsu Journal of Traditional Chinese Medicine (Jiang Su Zhong Yi Yao)	134 (0.3)	Chinese	No
Acupuncture Research (Zhen Ci Yan Jiu)	133 (0.3)	Chinese	Yes
Journal of Chinese Integrative Medicine (Zhong Xi Yi Jie He Xue Bao)	129 (0.3)	Chinese	Yes
Phytomedicine: International Journal of Phytotherapy and Phytopharmacology	128 (0.3)	English	Yes
Journal of Psychosomatic Research	124 (0.3)	English	Yes
Journal of the Korean Acupuncture & Moxibustion Society (Taehan Chimgu Hakhoe chi)	106 (0.2)	Korean	No
Total	8,637 (19.3)		

*Totals include named journals and any predecessor journals that were continued by the named journal.

The CAM register contains citations from 4,845 journals. Citations are quite concentrated in a few journals. One-third of the citations are found in the top 57 journals and two-thirds come from the top 420 journals. Among the 4,425 journals containing the remaining one-third of citations, 2,749/4,425 (62%) contributed only 1 or 2 citations to the register.

Of the 44,840 trial citations in the register, 93% were classified into one or more of the CAM intervention categories for which searches were conducted. The greatest concentrations were in non-vitamin, non-mineral dietary supplements (e.g., glucosamine, fish oil); Chinese herbal medicine (e.g., *Astragalus membranaceus*, *Schisandra chinensis*); diet-based therapies; vitamin and mineral interventions; and acupuncture (Table 4). The high representation of acupuncture and Chinese herbal medicine trial citations in the register might be explained by both a large number of trials being published in these areas as well as by the methods that we used to source the trial citations for the database (as described above).

**Table 4:** Number of CAM Field specialized register citations classified by type of CAM interventions

**CAM intervention**	**Citations *n *(% of citations in register)**	**MEDLINE-indexed citations N (% of citations in intervention category that are MEDLINE-indexed)**
Non-vitamin, non-mineral dietary supplements (e.g., glucosamine, fish oil)	15,140 (33.8)	12,529 (82.8)
Chinese herbal medicine (e.g., *Astragalus membranaceus*, *Schisandra chinensis*)	12,118 (27.0)	3,575 (29.5)
Diet-based therapies	9,009 (20.1)	9,009 (88.3)
Vitamin and mineral interventions (includes megavitamin therapies and vitamin or mineral therapies for other than medically diagnosed deficiencies or deficiency-related disorders)	7,741 (17.3)	6,468 (83.6)
Acupuncture	6,035 (13.5)	2,632 (43.6)
Relaxation (includes guided imagery and deep breathing)	3,743 (8.3)	3,194 (85.3)
Interventions using veritable energy modalities (unconventional uses of magnets, phototherapy, electrical stimulation, or ultrasonic therapy)	2,977 (6.6)	2,265 (76.1)
Chiropractic or osteopathic manipulation	2,606 (5.8)	2,041 (78.3)
Biofeedback	2,109 (4.7)	1,643 (77.9)
Massage	1,481 (3.3)	987 (66.6)
Traditional medicine not otherwise specified (e.g., Ayurveda, Kampo)	1,409 (3.1)	560 (39.7)
Meditation (includes mindfulness-based therapies)	1,259 (2.8)	1,056 (83.9)
Biologically based interventions not otherwise specified (e.g., balneotherapy, prolotherapy) and excluding interventions using energy fields	1,215 (2.8)	944 (77.7)
Interventions using putative energy fields (distant healing, prayer, qi gong, reiki, spiritual healing, and therapeutic touch)	1,210 (2.7)	801 (66.2)
Sensory art therapies (includes art, dance, drama, music, and play therapy)	1,136 (2.5)	911 (80.2)
Hypnosis	780 (1.7)	600 (76.9)
Homeopathy	755 (1.7)	302 (40.0)
Manipulative and body-based therapies not otherwise specified (e.g., Alexander technique, Pilates)	438 (0.9)	212 (48.4)
Yoga	333 (0.7)	242 (72.7)
Tai chi	188 (0.4)	149 (79.3)
Chelation therapy	153 (0.3)	148 (96.7)
Unclassified	3,093 (6.9)	2,124 (68.7)
Totals*	44,840	28,850 (64.3)

*Because individual citations were frequently classified into more than one CAM intervention category, the sum of the numbers of citations classified into each CAM intervention category exceeds the total number of citations in the CAM Field register (i.e., 44,840).

Of the 44,840 citations in the register, 85% were classified into 1 or more of the 25 categories of medical conditions. The greatest concentrations were in the categories of heart and circulation, anesthesia and pain, mental health, and endocrine and metabolic conditions (Table 5). Categories varied greatly in the proportion of citations included on MEDLINE, and the lowest percentage of MEDLINE-indexed citations was among citations not classified into any medical condition category. This is likely a result of non-MEDLINE citations being less likely to have abstracts or detailed keywords, and thus being less easy to categorize through searches.

**Table 5:** Number of CAM Field specialized register citations classified by type of medical condition

**Medical conditions**	**Citations *n *(% of citations in register)**	**MEDLINE-indexed citations N (% of citations in medical condition category that are MEDLINE-indexed)**
Heart and circulation	8,028 (17.9)	5,585 (69.6)
Anesthesia and pain control	7,656 (17.1)	5,492 (71.7)
Mental health	7,472 (16.7)	5,646 (75.6)
Endocrine and metabolic	6,188 (13.8)	3,797 (61.4)
Gastroenterology	3,378 (7.5)	2,354 (69.7)
Orthopedics and trauma	3,331 (7.4)	2,263 (67.9)
Cancer	2,983 (6.7)	2,205 (73.9)
Lungs and airways	2,545 (5.7)	1,828 (71.8)
Tobacco, drugs, and alcohol dependence	2,286 (5.1)	1,935 (84.6)
Neonatal care	2,252 (5.0)	1,924 (85.4)
Rheumatology	2,199 (4.9)	1,580 (71.9)
Infectious disease	2,080 (4.6)	1,128 (54.2)
Pregnancy and childbirth	2,050 (4.6)	1,642 (80.1)
Kidney disease	1,849 (4.1)	1,207 (65.3)
Neurology	1,697 (3.8)	1,289 (76.0)
Gynecology	1,598 (3.6)	1,142 (71.5)
Skin	1,187 (2.6)	746 (62.8)
Dentistry and oral health	1,123 (2.5)	896 (79.8)
Ear, nose, and throat	1,108 (2.5)	742 (67.0)
Eyes and vision	764 (1.7)	505 (66.1)
Urology	690 (1.5)	593 (85.9)
Wounds	667 (1.5)	505 (75.7)
Developmental, psychosocial, and learning problems	656 (1.5)	567 (86.4)
Blood disorders	596 (1.3)	435 (73.0)
Genetic disorders	214 (0.5)	148 (69.2)
Unclassified	6,635 (14.8)	3,298 (49.7)
Totals*	44,840	28,850 (64.3)

*Because individual citations were frequently classified into more than one medical condition category, the sum of the numbers of citations classified into each medical condition category exceeds the total number of citations in the CAM Field register (i.e., 44,840).

## Discussion

The CAM Field specialized register is an important resource for both MEDLINE and non-MEDLINE citations of CAM controlled trials. The prevalence of MEDLINE-indexed trial citations reflects the searches conducted in PubMed (and later in CENTRAL) for MEDLINE-indexed citations retrieved using the CAM on PubMed search strategy. We must therefore acknowledge the strides made in identification of both controlled trial and CAM citations by the US National Library of Medicine since the CAM Field specialized register was last examined in 1998 [5]. The large number of non-MEDLINE citations reflects searches of CENTRAL, which includes non-MEDLINE citations, and the efforts of CAM Field collaborators in China, Canada, Japan, and Korea, whose contributions to the specialized register were of predominantly non-MEDLINE citations. Overall, less than two-thirds of register citations are MEDLINE-indexed, and Sampson et al. concluded that with incomplete MEDLINE indexing of a body of literature, a specialized register was of particular utility [24].

Particular strengths of the register include citations of nutritional and supplement-related interventions, and traditional medicine. While citations of chelation therapy, nutrition and supplement-related interventions, sensory art therapies, relaxation, and meditation are likely to be MEDLINE-indexed (at least 80% of all these citations are indexed in MEDLINE), citations of traditional medicine interventions and homeopathy are less likely to be MEDLINE-indexed (fewer than 50% indexed in MEDLINE) and thus may be more difficult to locate. Therefore, the CAM Field specialized register may be a particularly useful resource for identifying citations of trials to be included in systematic reviews of traditional medicine interventions, particularly TCM. Similarly, the CAM Field specialized register may be a useful source of trials for systematic reviews covering CAM interventions for medical conditions in which a lower proportion of citations are MEDLINE-indexed (e.g., endocrine and metabolic conditions or infectious disease).

Among the 4,845 journals containing citations in the CAM Field register, 9% contain two-thirds of the register citations, and 57% contain only one or two register citations each. One unanswered question is whether this distribution of journals in the register represents the *true* distribution of CAM trials across journals or whether instead it is an artifact resulting from the way that the register was developed. There is no way to definitively answer this question because there exists no ‘gold standard’ complete database of CAM trials against which the journal distribution in the CAM Field register can be compared. However, we believe that the distribution of journals in the CAM Field register is largely an artifact of the way the register was developed. This is because, in identifying citations for register inclusion, contributors often searched bibliographic databases to identify trial citations on specific topics for their systematic reviews rather than comprehensively searching entire journals for all CAM trials. As a result, some journals for which only one or two CAM trial citations were identified for register inclusion may have many additional trials that have not yet been identified. Continued efforts to identify trials will likely change the distribution of citations across journals. Such efforts may also change the characteristics of the register in other ways (e.g., the number and proportion of trials covering particular CAM interventions or published in particular languages) that are not possible to quantify in advance. While the ultimate aim is for the CAM Field register to be a comprehensive source of CAM controlled trials, the register cannot currently be considered to be comprehensive. Therefore, systematic reviewers of CAM interventions should search multiple electronic and other sources for relevant CAM trials, in addition to searching the CAM Field register.

The strength of the register in terms of its coverage of difficult to locate trials may, however, be associated with potential weaknesses in terms of the quality of these trials. The largest subset of non-MEDLINE citations in the register (51%) is trials published in Chinese. These Chinese-language trials were included in the register if the trial publication stated that a random or quasi-random procedure was used to assign participants to treatment groups. However, a recent telephone survey of authors of ‘claimed’ randomized trials conducted in China discovered that only 7% could be confirmed to use a random method to assign participants to treatment groups [25]. Inclusion in systematic reviews of such Chinese trials claiming to be randomized, but not confirmed as such by systematic reviewers, may inflate these reviews’ meta-analytic effect estimates [26]. In addition, a 1998 review of the outcomes of non-English language trials by Vickers et al. found that acupuncture trials conducted in China reported positive results 100% of the time, and Chinese trials of other interventions reported positive results 99% of the time, strongly suggesting the preferential publication in China of trials with positive results [27]. Although Chinese language trials reflect the majority of non-English language trials included in the register, the issue of a publication or reporting bias favoring positive results may also be relevant to other non-English trials included in the register. For example, the Vickers et al. 1998 review found that not only acupuncture trials from China, but also acupuncture trials from Japan, Hong Kong, and Taiwan were uniformly positive. Also, a recent preliminary investigation into the results of the Japanese Kampo trials included in the CAM Field register found that only a small number of Kampo trials have negative results (Kiichiro Tsutani, personal communication, 14 March 2011), and an informal assessment of CAM Korean trials indicated that negative results were rare (Byeung-Cheul Shin, personal communication, 13 July 2012). The positive results of non-English language CAM trials likely explain why including such trials in CAM-related systematic reviews tends to inflate meta-analysis effect estimates, according to a 2005 empirical study [28].

It is possible that the methodological quality of the more recent Chinese-language trials may, however, be better than that of the earlier trials because, for example, the CONSORT statement has recently been more widely disseminated in China [29], including in Chinese journals of TCM [30]. In addition, while the Vickers et al. 1998 review [27] found that Chinese acupuncture trials published up to 1998 were uniformly positive, it is not known whether or not more recent Chinese trials are also likely to be uniformly positive. An important topic area for future research is to determine whether there is a publication bias favoring positive results in more recent trials from China. However, while conducting research studies to assess for the likelihood of publication bias in Chinese trials may be informative in determining the scope of the problem, the only way to avoid publication bias in Chinese trials is to ensure that all initiated Chinese language trials are known about through the registration of Chinese trials at inception, which is currently being implemented [31]. Universal trial registration, in conjunction with reporting of trial registration numbers in publications, might also serve as a tool in addressing duplicate publication [32], which some studies have observed to be prevalent among Chinese, Japanese, and Korean trials [33-35]. In the interim, for systematic reviews including a large number of Chinese trials, a possible approach for assessing the impact of a potential publication bias related to the Chinese trials may be to mark the Chinese trials in funnel plots in systematic reviews.

Despite these concerns over the validity of Chinese trials in general, it seems inappropriate to exclude trials from systematic reviews on the basis of language or country of publication alone. A more measured approach may be to search for Chinese trials and to telephone interview the authors of potentially eligible trials to try and assess whether the trials were truly randomized before including them in the review. If concerns about the validity of the trials remain, even after the telephone interviews with the authors, a possible approach is to include in the review those trials (Chinese or Western) for which there remains uncertainty about whether true randomization was used, but to be more restrictive when presenting the key findings, such as the abstract conclusions and the summary of findings table [36]. Another approach may be to analyze the potential influence of risk of bias measures (e.g., adequacy of randomization) on effect estimates using subgroup analyses or sensitivity analyses. Either way, such assessment and analysis approaches should probably be based on risk of bias measures rather than on the language of country of origin of the trials. This is because generalizing about *individual* Chinese language trials, for example, based on meta-research of the characteristics of Chinese trials *overall* would be an erroneous oversimplification. Instead, each trial included in a systematic review needs to be individually evaluated on its risks of bias, assuming either that the trial publication is sufficiently informative or that the trial author can be contacted for further information. Such risk of bias assessments can then be incorporated into the review’s analysis.

In addition to providing a source of trials for inclusion in systematic reviews, the register may also be used for investigations into the optimal use of CAM research resources and the prioritization of future CAM reviews. This analysis of the types of CAM interventions and health conditions covered in the register is a first step in conducting such investigations. That is, the number of citations related to various CAM interventions (e.g., diet-based therapies) and the number of citations related to various medical conditions (e.g., endocrine and metabolic disorders) may be used for research into whether there is a correlation between those CAM interventions most frequently investigated in trials and those most commonly used, and whether the most serious or prevalent health conditions are proportionately represented with the highest number of CAM trials. If trials of commonly used interventions and/or trials for serious or prevalent health conditions are lacking, this may indicate that CAM research resources should be directed to these areas. In addition, the trial database may also be useful for prioritizing future Cochrane reviews by identifying CAM intervention/health condition pairings for which there are available trials in the register but no existing Cochrane review. The fact that the same classification categories were used for CAM intervention types and health conditions, for both the trial citations in the specialized register and for a separate database of CAM-related Cochrane reviews [10], should facilitate such identification. However identifying potential future systematic reviews to prepare will require additional narrowing down of some of our CAM intervention type categories (e.g., “Chinese herbal medicine”) and health condition categories (e.g., “mental health”) in order to identify more specific intervention/condition pairings (e.g., the Chinese herbal medicine formula Free and Easy Wanderer for depression) for systematic reviews. Future plans for the CAM Field specialized register include augmenting the size and scope of the register through ongoing searches and international partnerships, and developing methods to characterize groups of trials according to intervention/condition characteristics and mapping these groups to gaps in Cochrane systematic reviews.

## Conclusions

The number of citations included in the CAM Field specialized register increased nearly tenfold between 2006 and 2011 as a result of a program of extensive searching and partnerships with international collaborators. Many CAM Field register citations are not MEDLINE-indexed and many of these non-MEDLINE-indexed citations are published in languages other than English. While the register provides access to thousands of difficult to locate citations of trials, many of these trials are likely to be of low quality and may overestimate treatment effects. When these trials are considered for inclusion in systematic reviews, it is extremely important that their risk of bias is adequately assessed.

## Competing interests

The authors declare that they have no competing interests.

## Authors’ contributions

Study concept and design: LSW, EM. Contribution of trial citations: JPB (citations of trials of Ayurveda and other CAM interventions included in the South Asian Database of Controlled Clinical Trials), KC (citations of Korean controlled trials of traditional medicine interventions), MSL (citations of Korean controlled trials of traditional medicine interventions), XL (citations of Chinese controlled trials of TCM interventions), JL (citations of Chinese controlled trials of TCM interventions), DM (citations of CAM controlled trials identified from specialized databases), TO (citations of Kampo controlled trials identified and evaluated by the Japan Society for Oriental Medicine), EP (citations of CAM-related controlled trials included in the African Trials Register), MS (citations of CAM controlled trials identified from specialized databases), B-CS (citations of Korean controlled trials of traditional medicine interventions), PT (citations of trials of Ayurveda and other CAM interventions included in the South Asian Database of Controlled Clinical Trials), KT (citations of Kampo controlled trials identified and evaluated by the Japan Society for Oriental Medicine). Data management: LSW, EM. Analysis of data: LSW, EM. Interpretation of data: EM, LSW, MS, DAvdW, LMB. Drafting the manuscript: Introduction, Methods, and Results: LSW, EM. Discussion: EM, LSW. Critically revised manuscript for important intellectual content and provided approval of the final manuscript: EM, LSW, MS, JPB, BMB, LMB, KC, MSL, XL, JL, DM, TO, EP, B-CS, PT, KT, and DAvdW. All authors read and approved the final manuscript.

## Supplementary Material

Additional file 1 Protocol for bibliometric analysis of the CAM Field specialized register. Click here for file

Additional file 2 List of therapies included as CAM. Click here for file

Additional file 3 CAM search strategies for CAM interventions. Click here for file

## References

[B1] Dickersin K Manheimer E Wieland S Robinson KA Lefebvre C McDonald S Development of the Cochrane Collaboration's CENTRAL Register of controlled clinical trials Eval Health Prof 2002 25 38 64 11868444 10.1177/016327870202500104

[B2] Egger M Juni P Bartlett C Holenstein F Sterne J How important are comprehensive literature searches and the assessment of trial quality in systematic reviews? Empirical study Health Technol Assess 2003 7 1 76 12583822

[B3] Hofmans EA Acupuncture and Medline Lancet 1990 336 57 1973242 10.1016/0140-6736(90)91574-t

[B4] Kleijnen J Knipschild P The comprehensiveness of Medline and Embase computer searches. Searches for controlled trials of homoeopathy, ascorbic acid for common cold and ginkgo biloba for cerebral insufficiency and intermittent claudication Pharm Weekbl Sci 1992 14 316 320 10.1007/BF01977620 1437515

[B5] Vickers AJ Bibliometric analysis of randomized trials in complementary medicine Complement Ther Med 1998 6 185 189 10.1016/S0965-2299(98)80026-5

[B6] Lefebvre C Manheimer E Glanville J Higgins JPT, Green S Chapter 6: Searching for studies Cochrane Handbook for Systematic Reviews of Interventions Version 5.1.0 [updated March 2011] 2011 The Cochrane Collaboration Available from http://www.cochrane-handbook.org (accessed 14 January 2013)

[B7] Committee on the Use of Complementary and Alternative Medicine by the American Public Chapter 5: State of emerging evidence on CAM Board of Health Promotion and Disease Prevention, Institute of Medicine of the National Academies. Complementary and Alternative Medicine in the United States 2005 Washington DC: The National Academies Press Available from http://books.nap.edu/catalog/11182.html (accessed 14 January 2013)

[B8] US National Center for Complementary and Alternative Medicine About CAM on PubMed Bethesda, MD: US Department of Health and Human Services. National Institutes of Health Available from http://nccam.nih.gov/research/camonpubmed (accessed 14 January 2013)

[B9] US National Library of Medicine Search strategy used to create the Complementary Medicine subset on PubMed Bethesda, MD: US Department of Health and Human Services. National Institutes of Health Available from http://www.nlm.nih.gov/bsd/pubmed_subsets/comp_med_strategy.html (accessed 14 January 2013)

[B10] Wieland LS Manheimer E Berman BM Development and classification of an operational definition of complementary and alternative medicine for the Cochrane collaboration Altern Ther Health Med 2011 17 50 59 *Both authors contributed equally to this work 21717826 PMC3196853

[B11] World Health Organization Traditional medicine, Fact Sheet No. 134 2008 Geneva: World Health Organization Available from http://www.who.int/mediacentre/factsheets/fs134/en/index.html (accessed 14 January 2013)

[B12] Cogo E Sampson M Ajiferuke I Manheimer E Campbell K Daniel R Moher D Searching for controlled trials of complementary and alternative medicine: a comparison of 15 databases Evid Based Complement Alternat Med 2011 2011 858246 Epub 2011 Jun 23 19468052 10.1093/ecam/nep038 PMC3137728

[B13] Kim KH Kong JC Choi JY Choi TY Shin BC McDonald S Lee MS Impact of including Korean randomized controlled trials in Cochrane reviews of acupuncture PLoS One 2012 7 e47619 10.1371/journal.pone.0047619 23071826 PMC3469498

[B14] Okabe T Tsutani K Evidence Reports of Kampo Treatment 2010:345 Randomized Controlled Trials (EKAT 2010) 2011 Task Force for Evidence Reports / Clinical Practice Guidelines (ER/CPG-TF), Special Committee for Evidence-based Medicine (EBM), The Japan Society for Oriental Medicine (JSOM) Available from http://www.jsom.or.jp/medical/ebm/ere/pdf.html (accessed 14 January 2013)

[B15] Barnabas J Yamuna BGC Parthasarathy V Venkatesh S Tharyan P Access to evidence from countries in South Asia: the South Asian Database of Controlled Clinical Trials and the South Asian Cochrane Network and Centre’s Digital Library17th International Cochrane Colloquium; 2009 Oct 11–14 Singapore Abstract no. P06-8. Available at http://www.imbi.uni-freiburg.de/OJS/cca/index.php?journal=cca&page=article&op=view&path%5B%5D=8010 (accessed 14 January 2013)

[B16] Pienaar E African Trials Register: 5 years later, where are we now13th International Cochrane Colloquium; 2005 Oct 22–26 Melbourne Abstract no. P021. Available at http://www.imbi.uni-freiburg.de/OJS/cca/index.php?journal=cca&page=article&op=view&path%5B%5D=1232 (accessed 14 January 2013)

[B17] Siegfried N Busgeeth K Clarke M Pienaar E Volmink L African randomized controlled trials - too hard to find14th International Cochrane Colloquium; 2006 October 23–26 Dublin Abstract no. P017. Available at http://www.imbi.uni-freiburg.de/OJS/cca/index.php?journal=cca&page=article&op=view&path%5B%5D=1972 (accessed 14 January 2013)

[B18] Barnes PM Bloom B Nahin RL Complementary and alternative medicine use among adults and children: United States Natl Health Stat Report 2008 2007 1 23 19361005

[B19] Sampson M McGowan J Cogo E Grimshaw J Moher D Lefebvre C An evidence-based practice guideline for the peer review of electronic search strategies J Clin Epidemiol 2009 62 944 952 10.1016/j.jclinepi.2008.10.012 19230612

[B20] The Cochrane Library Independent high quality evidence for healthcare decision making John Wiley & Sons, Ltd Available from http://www.thecochranelibrary.com (accessed 14 January 2013)

[B21] Cochrane Central Register of Controlled Trials (CENTRAL) Issue 1, part of The Cochrane Library 2012 http://www.thecochranelibrary.com

[B22] Clarke M Clarke T The Cochrane Central Register of Controlled Trials and the contribution from the specialized registers of Cochrane Review Groups A project funded in part by a grant from The Cochrane Collaboration’s Discretionary Fund 2008

[B23] Tang JL Zhan SY Ernst E Review of randomised controlled trials of traditional Chinese medicine BMJ 1999 319 160 161 10.1136/bmj.319.7203.160 10406751 PMC28166

[B24] Sampson M Daniel R Cogo E Dingwall O Sources of evidence to support systematic reviews in librarianship J Med Libr Assoc 2008 96 66 69 10.3163/1536-5050.96.1.66 18219385 PMC2212322

[B25] Wu T Li Y Bian Z Liu G Moher D Randomized trials published in some Chinese journals: how many are randomized? Trials 2009 10 46 10.1186/1745-6215-10-46 19573242 PMC2716312

[B26] Savovic J Jones HE Altman DG Harris RJ Juni P Pildal J Als-Nielsen B Balk EM Gluud C Gluud LL Influence of reported study design characteristics on intervention effect estimates from randomized, controlled trials Ann Intern Med 2012 157 429 438 22945832 10.7326/0003-4819-157-6-201209180-00537

[B27] Vickers A Goyal N Harland R Rees R Do certain countries produce only positive results? A systematic review of controlled trials Control Clin Trials 1998 19 159 166 10.1016/S0197-2456(97)00150-5 9551280

[B28] Pham B Klassen TP Lawson ML Moher D Language of publication restrictions in systematic reviews gave different results depending on whether the intervention was conventional or complementary J Clin Epidemiol 2005 58 769 776 10.1016/j.jclinepi.2004.08.021 16086467

[B29] MacPherson H Altman DG Improving the quality of reporting acupuncture interventions: describing the collaboration between STRICTA, CONSORT and the Chinese Cochrane Centre J Evid Based Med 2009 2 57 60 10.1111/j.1756-5391.2009.01008.x 21348986

[B30] Fei YT Liu JP Improving the quality of reporting Chinese herbal medicine trials: an elaborated checklist J Chinese Integr Med (Zhong Xi Yi Jie He Xue Bao) 2008 6 233 238 10.3736/jcim20080302 18334139

[B31] Wu T Li Y Liu G Li J Wang L Du L Chinese clinical trial registry: mission, responsibility and operation J Evid Based Med 2011 [ Epub ahead of print]onlinelibrary.wiley.com/doi/10.1111/j.1756-5391.2011.01137.x/pdf 10.1111/j.1756-5391.2011.01137.x 21894612

[B32] Antes G Dickersin K Trial registration to prevent duplicate publication JAMA 2004 291 2432 15161892 10.1001/jama.291.20.2432-c

[B33] Errami M Garner H A tale of two citations Nature 2008 451 397 399 10.1038/451397a 18216832

[B34] Kitagawa M Tsutani K Duplicate publication cases in the field of Kampo (Japanese herbal medicine) in Japan J Chinese Integr Med (Zhong Xi Yi Jie He Xue Bao) 2011 9 1055 1060 10.3736/jcim20111003 22015184

[B35] Kim SY Hahm CK Bae CW Cho HM Duplicate publications in Korean medical journals indexed in Korea Med J Korean Med Sci 2008 23 131 133 10.3346/jkms.2008.23.1.131 18303213 PMC2526492

[B36] Deeks JJ Higgins JPT Altman DG Higgins JPT, Green S Chapter 9: Analysing data and undertaking meta-analyses Cochrane Handbook for Systematic Reviews of Interventions. Version 5.1.0 [updated March 2011] 2011 The Cochrane Collaboration Available from http://www.cochrane-handbook.org (accessed 14 January 2013)

