# Supplementary material for: Bibliometric and content analysis of the Cochrane Complementary Medicine Field specialized register of controlled trials
Source: Syst Rev. 2013 Jul 4;2:51. doi: 10.1186/2046-4053-2-51 (PMC3704678; doi:10.1186/2046-4053-2-51)
Supplement: Additional file 1 — Protocol for bibliometric analysis of the CAM Field specialized register. [file 2046-4053-2-51-S1.pdf]

Protocol for Bibliometric Analysis of the Cochrane Complementary Medicine Field Specialized Register

1.1 Title: Bibliometric Analysis of the Cochrane Complementary Medicine Field Specialized Register

1.2 Research team:

Principal investigators:[1]

L. Susan Wieland, MPH, PhD [University of Maryland School of Medicine, Center for Integrative Medicine; Methodologist, Cochrane CAM Field]

Eric Manheimer, MS [University of Maryland School of Medicine, Center for Integrative Medicine; Administrator and Methodologist, Cochrane CAM Field]

Co-investigators:[2]

Jabez Paul Barnabas [Christian Medical College, Vellore, India; Administrator and Trials Search Coordinator, South Asian Cochrane Center; *contribution to the specialized register*: trials of Ayurveda, Unani and other traditional medicines of South Asia]

Lex Bouter, PhD [VU University Amsterdam; *contribution to the project/paper*: methodological support and guidance]

Kiho Cho, OMD, PhD [Kyunghee University; *contribution to the specialized register*: trials of Korean medicine]

Myeong Soo Lee, MSc, PhD [Korea Institute of Oriental Medicine; *contribution to the specialized register*: trials of Korean medicine]

---

[1] In terms of author order of the resulting publication, we have proposed that Susan Wieland and Eric Manheimer be first and second authors respectively, Brian Berman be last author, and the remaining co-investigators be ordered alphabetically in the author list.

[2] Most authors are long-term and senior Cochrane contributors. Their contribution in terms of trial identification or methodological support is listed for each author. Lex Bouter and Danielle van der Windt are involved in this project primarily to provide methodological support and guidance, particularly because this bibliometric analysis is one of the papers included in Eric Manheimer's PhD dissertation, under the supervision of Lex, Danielle, and Brian. Lex and Danielle are both long-term contributors to the Cochrane Collaboration, each having published multiple Cochrane reviews. Lex was also the former coordinating editor of the Cochrane Back Review Group, prior to his appointment as the President and Vice-Chancellor of VU University, Amsterdam.

Xun Li, PhD student of Professor Jianping Liu [Beijing University of Chinese Medicine, Center for Evidence-Based Chinese Medicine; *contribution to the specialized register*: Chinese language trials of TCM]

Jianping Liu, MD [Beijing University of Chinese Medicine, Center for Evidence-Based Chinese Medicine; *contribution to the specialized register*: Chinese language trials of TCM]

David Moher, MSc, PhD [Ottawa Health Research Institute; *contribution to the specialized register*: difficult to locate CAM trials identified through searches of specialized electronic bibliographic databases]

Tetsuro Okabe, MD [University of Tokyo; *contribution to the specialized register*: Japanese Kampo trials]

Elizabeth D. Pienaar [South African Cochrane Centre; *contribution to the specialized register*: traditional African medicine trials]

Margaret Sampson, MLIS, PhD [Children's Hospital of Eastern Ontario; *contribution to the specialized register*: difficult to locate CAM trials identified through searches of specialized electronic bibliographic databases; information scientist for assessment and manipulation of records in specialized register for this publication]

Byung-Cheul Shin, OMD, PhD [Pusan National University; *contribution to the specialized register*: trials of Korean medicine]

Prathap Tharyan, MD [Christian Medical College, Vellore, India; Director, South Asian Cochrane Centre; *contribution to the specialized register*: trials of Ayurveda, Unani and other traditional medicines of South Asia]

Kiichiro Tsutani, MD, PhD [University of Tokyo; Director of the Japanese Network for the Cochrane Collaboration; *contribution to the specialized register*: Japanese Kampo trials]

Danielle van der Windt, MSc, PhD [Keele University; *contribution to the project/paper*: methodological support and guidance]

Brian Berman, MD [University of Maryland School of Medicine, Center for Integrative Medicine; Director, Cochrane CAM Field]

1.3 Protocol details: Version 1.0

August 31, 2011

2. List of Acronyms: CAM Field = Cochrane Complementary Medicine Field

RCT= Randomized Controlled Trial

3. Summary

This document is a protocol for a bibliometric analysis of the CAM Field specialized register, which is the most comprehensive database available of information on controlled clinical trials of CAM therapies. The register includes difficult to locate English language trials, as well as non-English trials of traditional medicine therapies contributed by Cochrane collaborators from around the world. It is a valuable source of information for researchers conducting systematic reviews on CAM therapies, and also for practitioners, consumers, policy makers and researchers seeking information about the current status of CAM research. Our primary objective is to describe the key characteristics of the CAM Field specialized register and the trials contained therein.

4. Background

Complete identification of relevant trial evidence is an essential step in conducting a systematic review, and finding and collecting trial reports has been an aim of the Cochrane Collaboration from its inception. As part of this mission, the Collaboration developed the Cochrane Central Register of Controlled Trials (CENTRAL), a searchable database of reports of controlled trials. The Collaboration has agreements with the publishers of MEDLINE and EMBASE, ensuring that all citations of controlled trials from those databases are republished in CENTRAL. Cochrane entities also contribute citations of controlled trials to CENTRAL. Cochrane Review Groups and, optionally, Cochrane Fields, are responsible for identifying controlled trials in their area of health care, collecting those trials in databases (called 'specialized registers'), and contributing the specialized registers to CENTRAL. Cochrane Review Groups are also expected to contribute any controlled trials they find that are not in their area of health care to CENTRAL. These records, which are not in specialized registers, are called 'handsearch records'. The 30 Cochrane Centers and Branches around the world have as one of their functions the identification of trial reports published in their geographic regions, and they are also expected to contribute any identified trial reports to CENTRAL as handsearch records. Because CENTRAL includes trial reports from multiple sources, including not only

MEDLINE and EMBASE but also regional and subject-specific databases and citations to trial reports not included in databases, CENTRAL is considered to be the best single source of reports of controlled trials for inclusion in systematic reviews.

The complete identification of trial evidence can be particularly challenging for systematic reviews of CAM therapies. The disadvantage of relying exclusively upon sources such as MEDLINE for trial identification is illustrated by research conducted by Egger et al. Egger and colleagues (Egger et al 2003) analyzed the characteristics of trials included in a group of meta-analyses, including both conventional and CAM-related meta-analyses. They found that in CAM-related meta-analyses, the proportion of non-MEDLINE-indexed trials (40.9%) was approximately twice that proportion seen in conventional medicine meta-analyses (20.5%). If authors had searched only MEDLINE for trials, they would have missed many trials, and the proportion missing in CAM reviews would have been even worse than the proportion missing from conventional medicine reviews. Earlier research using various gold standards of known trials for specific CAM therapies found that the percentage of known trials included in MEDLINE was 58% for acupuncture trials (Hofmans et al 1990), 31% for ginkgo trials (Kleijnen et al 1992), and 17% for homeopathy trials (Kleijnen et al 1992). Ensuring that CENTRAL contains both MEDLINE and non-MEDLINE citations to CAM-related controlled trials is therefore important for the unbiased conduct of Cochrane systematic reviews on CAM therapies.

The CAM Field maintains a specialized register of controlled trials of CAM therapies which is regularly submitted to CENTRAL. In 1998, a bibliometric analysis of the CAM Field specialized register described the database as containing 3774 randomized controlled trials (Vickers 1998). In 2007, the CAM Field began an active program to update and enlarge the CAM Field specialized register. Efforts focused on 1) thorough identification of all CAM-related trials in MEDLINE by means of the CAM on PubMed subset, a search strategy that was jointly developed by the US National Library of Medicine (NLM) and National Center for Complementary and Alternative Medicine (NCCAM) and introduced in PubMed in 2001; 2) identification and retrieval of CAM-related trials present in CENTRAL and not already included in the CAM Field specialized register; 3) identification of difficult-to-find CAM-related controlled trials through searching of CAM-specific electronic databases; and 4) identification of controlled

trials in traditional medicine, including Chinese-language trials of CAM therapies, conducted through partnerships with international collaborators. As a result of these efforts, there are a total of 39,144 CAM Field specialized register records in CENTRAL Issue 4, 2010.

5. Specific aims of study

The primary aim is to describe the current contents of the Cochrane CAM Field specialized register of trials.

6. Study design

This is a bibliometric analysis.

7. Study group

7.1 Inclusion criteria: All records in the CAM Field specialized register meet the following two criteria for inclusion: 1) they are reports of controlled clinical trials, and 2) they are CAM-related.

1. *Reports of controlled clinical trials*

We considered controlled clinical trials to be trials meeting the inclusion criteria for CENTRAL that were formulated and agreed upon in November 1992 and are published in Chapter 6.3 of the Cochrane Handbook (see Box 1). MEDLINE records that are indexed as human studies and also indexed with the publication type terms 'Randomized Controlled Trial' or 'Controlled Clinical Trial' are automatically downloaded into CENTRAL four times per year as part of the regular building of CENTRAL. They are therefore also considered controlled trials for purposes of CENTRAL and the CAM Field specialized register. We note, however, that the US NLM definitions of 'Randomized Controlled Trial' and 'Controlled Clinical Trial' are not identical to the Cochrane criteria for RCT and CCT (Box 2). The NLM definition of the 'Controlled Clinical Trial' publication type in MEDLINE, which includes control groups based on historical comparisons, does

not meet the inclusion criteria for CENTRAL. Since there is no mechanism to separate out studies using historical comparisons from other MEDLINE records using the 'Controlled Clinical Trial' publication type, either during the building of CENTRAL or internally within the CAM Field building of the CAM Field specialized register, it is possible that some records in CENTRAL and the CAM Field specialized register are based on historical comparisons and are not technically controlled clinical trials according to the CENTRAL definition.

For trials originating outside CENTRAL, we relied upon our collaborators to correctly classify records as reports from controlled trials according to Cochrane criteria. If we observed records that were clearly not reports from trials, we removed the records from the submission and contacted the collaborators to ensure that only controlled trials were sent in future.

2. Reports of CAM-related trials

We relied upon the CAM on PubMed search strategy to retrieve trials from MEDLINE and CENTRAL (see description of searches under 'Identification of sample' below). Records retrieved through use of this strategy were initially considered to be CAM-related.

For trials originating outside MEDLINE and CENTRAL, we considered trials to be CAM-related if they described therapies that were outside the practices and theories of disease and healing that are intrinsic to the conventional Western medical model.(cite IOM 2005). In some cases (e.g., acupuncture, homeopathy), this criterion was sufficient to indicate that a therapy was CAM. In other cases (e.g., vitamin supplementation), some uses of the therapy are accepted by the conventional Western model and others are not. CAM Field staff had confronted this issue when identifying Cochrane systematic reviews related to CAM, and had developed an operational definition of CAM-related

systematic reviews (Wieland, Manheimer & Berman). We followed these same operational criteria in classifying trials as CAM or non-CAM for the CAM Field specialized register. Some of the major decisions about the scope of CAM were: we excluded vitamins and other supplements that are administered parenterally in hospital settings, we excluded dietary supplementation for treatment or prevention of medically diagnosed deficiency states or disorders, we excluded vitamin supplements for preventing or treating disease in countries where vitamin deficiency is widespread, we excluded exercise therapies, with the exception of mind body exercise (e.g., yoga), and we excluded conventional psychotherapies. A full description of the CAM Field operationalization of CAM is available online at http://www.compmed.umm.edu/Camdef.asp. We conveyed this operational definition of CAM to our collaborators identifying trials outside the arena of traditional medicine, to ensure that only CAM trials were forwarded to the CAM Field.

7.2 Sample size: The total number of CAM Field specialized register records in CENTRAL is 39,144 records as of Issue 4, 2010. The CAM Field specialized register at the CAM Field base currently contains 43,726 records, which have been submitted for publication in CENTRAL Issue 4, 2011. After all contributors have sent their records to the CAM Field, and all outstanding records have been added to the CAM Field specialized register, the register will undergo final checking and deduplication. The database will then be ‘frozen’ for analysis.

8. Identification of sample

We will include all records in the Cochrane CAM Field specialized register as of [date that database is ‘frozen’].

Brief description of how records have been/will be sourced:

1) CAM Field staff began conducting quarterly searches of MEDLINE in 2006, using the CAM on PubMed search strategy and eliminating from search results 1) records not indexed with the RCT publication type, and 2) records indexed with the Medical Subject Heading (MeSH) for animals and not the MeSH for humans (in order to eliminate animal-only studies). In 2008, an information specialist translated the CAM on PubMed search strategy into a format for use in the Wiley interface of CENTRAL, and beginning later that year, searches in PubMed were stopped and replaced with quarterly searches of CENTRAL. Restrictions to RCT publication status were no longer used, as CENTRAL contains both RCT and CCT records, and not all records in CENTRAL are clearly designated as one or the other. Were search retrievals to be restricted to records clearly labeled as RCT, some RCT records would be omitted from the specialized register. Restrictions to human studies were also no longer incorporated in the search strategy, as CENTRAL contains only studies in humans. In addition to MEDLINE records, CENTRAL contains controlled trial records automatically downloaded from EMBASE, controlled trial records submitted from Cochrane Review Group specialized registers, and controlled trial records submitted to CENTRAL as handsearch results, and therefore a search of CENTRAL identifies more trials than a search of MEDLINE alone. A limitation of searches of non-MEDLINE, non-EMBASE records in CENTRAL is that records from other sources do not include abstracts due to copyright restrictions. The advantages of searching CENTRAL and not searching MEDLINE on PubMed were primarily that there was no need to deduplicate results between CENTRAL and MEDLINE on PubMed, and the risk of including duplicate records in the CAM Field specialized register was reduced.
2) External searches consisting of
    a. Records submitted to the CAM Field by the Beijing University of Chinese Medicine (BUCM) under the direction of Professor Jianping Liu. Beginning in 2008, staff at BUCM searched electronic databases and handsearched Traditional Chinese Medicine (TCM) journals for controlled trials of TCM therapies. All citations were translated into English, entered into a ProCite reference management database, and submitted to the CAM Field for inclusion in the CAM Field specialized register. A total of 6000 records have been submitted to the CAM

Field to date, including 1792 records in September 2008, 2470 records in December 2009, and 1738 records in May 2011. Efforts to identify additional TCM trials are ongoing.

b. Records submitted to the CAM Field by researchers at the Ottawa Hospital Research Institute (OHRI) (formerly headquartered at the Chalmers Research Group, CHEO Research Institute) under the direction of Professor David Moher. Information specialists searched several small CAM-specific databases for difficult-to-identify controlled trials of CAM interventions (ref Cogo?). All citations were imported into a ProCite database, information about the source database and the type of CAM intervention was included for each citation, and the database was submitted to the CAM Field for inclusion in the CAM Field specialized register. A total of 3338 records have been submitted to the CAM Field to date, including 2504 records submitted in April 2008 and 834 records submitted in September 2010.

c. Records identified through the efforts of the Special Committee for Evidence Based Medicine (EBM) of the Japan Society for Oriental Medicine (JSOM) to collect and critique controlled trial evidence on Kampo therapies. As of 2010, 345 randomized controlled trials of Kampo therapies were identified, structured abstracts were prepared for each trial, and the citations and structured abstracts were published online in English. In March 2011, one of the leaders of this initiative, Professor Kiichiro Tsutani of the University of Tokyo, met with CAM Field staff at the Center for Integrative Medicine. During this meeting he agreed to allow CAM Field staff to incorporate the citations associated with these 345 trials into the CAM Field specialized register. In April 2011, 352 citations associated with the 345 Kampo RCTs identified through 2010 were added to the CAM Field specialized register, together with links to the online structured abstracts. Efforts to identify and critique RCTs of Kampo therapies are ongoing, and it is expected that additional citations will be added to the CAM Field specialized register when additional RCTs are available.

d. Records submitted to the CAM Field by researchers at the Korea Institute of Oriental Medicine, under the direction of Dr. Myeong Soo Lee. Controlled trials of traditional

medicine therapies conducted in Korea and not published in MEDLINE journals were identified by handsearchers. Citations were translated into English and entered into a ProCite database, and the records were submitted to the CAM Field. In October 2010, 123 records were submitted to the CAM Field for inclusion in the specialized register. Efforts to identify Korean trials of traditional medicine are ongoing.

e. Records of South Asian trials of Ayurveda and other traditional CAM therapies, identified through searching the South Asian Database of Controlled Clinical Trials. Citations will be downloaded into a reference management database and sent to the CAM Field for inclusion in the CAM Field specialized register.

f. Records of African trials of CAM therapies, identified through searches conducted by Elizabeth Pienaar, information specialist at the South African Cochrane Center. Identified citations were entered into a ProCite database and forwarded to the CAM Field. In March 2011, 35 records were sent to the CAM Field for inclusion in the CAM Field specialized register.

9. Data

9.1 Data from the CAM Field specialized register to be collected/analyzed

- The number of records published in each calendar year
- The number of records published in each language, including English
- The number of records related to various CAM topic areas (e.g., acupuncture, homeopathy) (see Box 3)
- The number of records related to various medical conditions (e.g., arthritis, pain) (see Box 4)
- The number of records not available through MEDLINE/PubMed
- The number of records that are published in journals and the number of records that are either unpublished or published in non-journal sources
- The total number of journals represented in the register
- The journals in the register publishing the most CAM trials:

    - The conventional medicine journals publishing the most CAM trials (top 50)
    - The CAM journals publishing the most CAM trials(top 50) (see Box 5)
- The total number of CAM SR records contributed by each of the groups collaborating on this analysis

For the purposes of this study, we will assume all CAM Field specialized register records are RCT or CCT, as the specialized register records originate from PubMed searches for MEDLINE publication types RCT and CCT, from searches of CENTRAL, or from handsearching contributions of controlled trials to the CAM Field. Each one of those sources should produce records that are RCT or CCT, and eligible for CENTRAL. Therefore we do not plan to classify records in the CAM Field specialized register by RCT/CCT vs. other study design.

For the purposes of this paper, we will not distinguish between RCT and CCT records, for several reasons. First of all, the classification as RCT or CCT is based upon the judgment of the handsearcher (for records classified by Cochrane contributors) or indexer (for records classified according to publication type by NLM), using the best available information from the publication. The information available to different handsearchers, and their judgment about the record status as RCT or CCT, may vary from person to person. Against this background of individual variability, it is also possible that MEDLINE indexer classification codes for RCT and CCT also differ somewhat from Cochrane handsearcher judgments, in terms of sensitivity. Checking the decisions previously made for each record would be a large investment of time and possibly introduce additional error. Although most records in the CAM Field specialized register have been indexed or otherwise classified as RCT, CCT, or both, we have therefore chosen not to report classifications of records in the CAM Field specialized register by RCT vs. CCT.

9.2 Data handling

The register will be cleaned in preparation for analyses. The following steps have been or will be carried out:

1) Spot checking of records to identify clearly non-CAM or non-controlled trial citations was carried out by LS Wieland. This examination was carried out on all records submitted by collaborators, and is carried out both retrospectively and prospectively upon all CAM Field specialized register records retrieved from

searches of MEDLINE/PubMed and CENTRAL. The examination consists of visually inspecting the titles or abstracts of each record if the group of records checked is relatively small (under 500 records). The examination consists of database text word searches and spot checks if the group of records checked is large. For example, we realized that some collaborators were sending records for systematic reviews, and the phrase “systematic OR SR” was therefore searched in the record submission, and the titles and abstracts of all retrieved records were reviewed. As another example, we realized that the CAM on PubMed search strategy sometimes retrieves non-CAM trials of chemotherapy agents, and the term “chemotherapy” was therefore searched in the CAM Field specialized register, and the titles of all retrieved records were reviewed. Unfortunately, the size of the CAM Field specialized register precludes checking the title and abstract of every individual record.

2) Deduplication of records has been carried out by LS Wieland and will be checked using automated methods by M Sampson. Note that whenever duplicates are detected, and one of the records is a MEDLINE record, any information from the non-MEDLINE record will be copied to the MEDLINE record, the non-MEDLINE record will be deleted, and the MEDLINE record will be retained.
3) Identification of records present in MEDLINE has been carried out by LS Wieland and will be checked using batch citation methods by M Sampson. All journals in the CAM Field specialized register have been checked against the NLM Catalog for indexing in MEDLINE. If journals were indexed in MEDLINE but the individual record did not have a PubMed identifier (PMID), MEDLINE/PubMed was checked for the individual reference, and if the reference was found, the PMID was added to the record, causing the record to be classified as a MEDLINE record.
4) Identifying languages of publication for records not in English was carried out by LS Wieland. All MEDLINE journals in the CAM Field specialized register were checked against the NLM Catalog for language of publication. If articles in a journal were published in a language other than English, and there was only one non-English language of publication, the identifier for that non-English language was added to each of the records associated with that journal.

5) Conforming of journal names was carried out by LS Wieland and will be augmented by automated methods by M Sampson. The CAM Field specialized register was sorted by journal name and the results were visually inspected. Journal titles that were clearly variants (e.g., abbreviations, variations in punctuation) were conformed to the full journal name.

10. Limitations

One limitation of the study is that we have relied heavily on MEDLINE to identify controlled trials of interventions. We know that there are non-trials in MEDLINE that have been assigned the publication type RCT or CCT, and are mistakenly included in CENTRAL and therefore possibly in the CAM Field specialized register. We also recognize that there are controlled trials in MEDLINE that have not been assigned the publication type RCT or CCT and therefore are not automatically downloaded into CENTRAL, and may be missing from the CAM Field specialized register.

A second limitation of the study is that the CAM on PubMed search strategy does not produce results that correspond exactly to the CAM Field operationalization of CAM. We attempted to address this limitation by performing checks of search results to identify records that were clearly non-CAM, however we cannot be sure that all non-CAM records were found by these checks. In general, the result of this limitation is that the contents of the CAM Field specialized register may be overinclusive rather than underinclusive.

11. Statistical analysis

We will calculate frequencies.

12. Reporting and dissemination

We plan to submit a journal article for publication in the journal *Trials*.

To ascertain the reporting guidelines for protocols and final publications of bibliometric analyses, we checked the Equator website (www.equator.com) and consulted with experts in bibliometric analyses. We were unable to find any guidelines specific to bibliometric analyses, and to the best of our knowledge, reporting guidelines for

protocols and final publications of bibliometric analyses do not exist. We therefore used the outline of suggested items in a protocol for an observational study (found at http://www.ucl.ac.uk/joint-rd-unit/statistics/obs_protocol_guidelines.pdf) as a model for this protocol. For the final publication of this paper, we plan to use the STROBE guidelines (http://www.strobe-statement.org), adapted as appropriate.

Box 1.

Records identified for inclusion should meet the eligibility criteria devised and agreed in November 1992, which were first published, in 1994, in the first version of the *Handbook* (see Chapter 1, Section 1.4). According to these eligibility criteria:

A trial is eligible if, on the basis of the best available information (usually from one or more published reports), it is judged that:

- the individuals (or other units) followed in the trial were definitely or possibly assigned prospectively to one of two (or more) alternative forms of health care using

- random allocation or
- some quasi-random method of allocation (such as alternation, date of birth, or case record number).

Trials eligible for inclusion are classified according to the reader's degree of certainty that random allocation was used to form the comparison groups in the trial. If the author(s) state explicitly (usually by some variant of the term 'random' to describe the allocation procedure used) that the groups compared in the trial were established by random allocation, then the trial is classified as a RCT (randomized controlled trial). If the author(s) do not state explicitly that the trial was randomized, but randomization cannot be ruled out, the report is classified as a CCT (controlled clinical trial). The classification CCT is also applied to quasi-randomized studies, where the method of allocation is known but is not considered strictly random, and possibly quasi-randomized trials. Examples of quasi-random methods of assignment include alternation, date of birth, and medical record number.

The classification as RCT or CCT is based solely on what the author has written, not on the reader's interpretation; thus, it is not meant to reflect an assessment of the true nature or quality of the allocation procedure. For example, although 'double-blind' trials are nearly always randomized, many trial reports fail to mention random allocation explicitly and should therefore be classified as CCT.

Relevant reports are reports published in any year, of studies comparing at least two forms of health care (healthcare treatment, healthcare education, diagnostic tests or techniques, a preventive intervention, etc.) where the study is on either living humans or parts of their body or human parts that will be replaced in living humans (e.g., donor kidneys). Studies on cadavers, extracted teeth, cell lines, etc. are not relevant. *Searchers should identify all controlled trials meeting these criteria regardless of relevance to the entity with which they are affiliated.*

The highest possible proportion of all reports of controlled trials of health care should be included in CENTRAL. Thus, those searching the literature to identify trials should give reports the benefit of any doubts. Review authors will decide whether to include a particular report in a review.

Source: *The Cochrane Handbook for Systematic Reviews of Interventions*, Box 6.3.a: Cochrane definitions and criteria for randomized controlled trials (RCTs) and controlled clinical trials (CCTs)

Box. 2 U.S. NLM definitions for ‘Randomized Controlled Trial’ and ‘Controlled Clinical Trial’ publication types in MEDLINE

Randomized Controlled Trial

Work consisting of a clinical trial that involves at least one test treatment and one control treatment, concurrent enrolment and follow-up of the test- and control-treated groups, and in which the treatments to be administered are selected by a random process, such as the use of a random-numbers table.

Controlled Clinical Trial

Work consisting of a clinical trial involving one or more test treatments, at least one control treatment, specified outcome measures for evaluating the studied intervention, and a bias-free method for assigning patients to the test treatment. The treatment may be drugs, devices, or procedures studied for diagnostic, therapeutic, or prophylactic effectiveness. Control measures include placebos, active medicine, no-treatment, dosage forms and regimens, historical comparisons, etc. When randomization using mathematical techniques, such as the use of a random-numbers table, is employed to assign patients to test or control treatments, the trial is characterized as a ‘Randomized Controlled Trial’.

Source: *The Cochrane Handbook for Systematic Reviews of Interventions*, Box 6.3.b: US National Library of Medicine 2008 definitions for the Publication Type terms ‘Randomized Controlled Trial’ and ‘Controlled Clinical Trial’

Box. 3 Categories of CAM interventions used to characterize trials included in the CAM Field specialized register

Categories of CAM interventions were based upon the categories in the CAM Field topics list for Cochrane reviews of CAM interventions (http://www2.cochrane.org/reviews/en/subtopics/22.html) as well as other classifications of CAM therapies (e.g., the classifications of CAM therapies on the 2007 NHIS survey of use of CAM in the United States (cite Barnes)). Decisions about classification of CAM therapies are necessarily somewhat subjective, as many CAM therapies could be classified under more than one category (e.g., acupuncture is a traditional medical therapy that is also based upon putative engery fields). For this bibliometric analysis, the types of CAM interventions are classified as follows:

- Therapies from alternative medical systems
  - Acupuncture
  - Chinese herbal medicine
  - Homeopathy
  - Traditional medicine not otherwise specified (e.g., Ayurveda, Kampo)
- Biologically based therapies
  - Chelation therapy
  - Diet-based therapies (e.g., vegetarian diets)
  - Non-vitamin, non-mineral dietary supplements and herbal products (e.g., probiotics, ginseng)
  - Vitamin and mineral therapies (includes megavitamin therapies and vitamin or mineral therapies for other than medically diagnosed deficiencies or deficiency-related disorders)
  - Biologically based therapies not otherwise specified and excluding therapies using energy fields (e.g., balneotherapy, prolotherapy)
- Manipulative and body based therapies
  - Chiropractic or osteopathic manipulation
  - Massage
  - Manipulative and body based therapies not otherwise specified (e.g., Alexander technique, Pilates, reflexology)
- Mind-body therapies
  - Biofeedback
  - Hypnosis
  - Meditation (includes mindfulness-based therapies)
  - Relaxation (includes guided imagery and deep breathing)
  - Sensory art therapies (includes art, dance, drama, music, and play therapy)
  - Tai chi
  - Yoga
- Energy therapies
  - Therapies using putative energy fields (distant healing, prayer, qi gong, reiki, spiritual healing, and therapeutic touch)
  - Therapies using veritable energy modalities (unconventional uses of magnets, phototherapy, electrical stimulation, or ultrasonic therapy)

Box. 4 Categories of medical conditions used to characterize trials included in the CAM Field specialized register

Categories of medical conditions were based upon the browse list on the home page of the Cochrane Library (thecochranelibrary.com). The Cochrane Library browse list is primarily based upon medical conditions, although there are some categories related to populations (child health), interventions (complementary medicine), or other areas covered by Cochrane reviews (consumer & communication strategies, effective practice/health systems, methodological & diagnostic, & public health). We chose to use the medical conditions from the Cochrane Library browse list because this is a scheme already used within Cochrane to classify Cochrane reviews, and using similar categories for Cochrane reviews and controlled trials may provide a beginning approach to outlining relationships between reviews and trials. The categories are as follows:

Anesthesia & pain control
Blood disorders
Cancer
Dentistry & oral health
Developmental, psychosocial, & learning problems
Ear, nose, & throat
Endocrine & metabolic
Eyes & vision
Gastroenterology
Genetic disorders
Gynecology
Heart & circulation
Infectious disease
Kidney disease
Lungs & airways
Mental health
Neonatal care
Neurology
Orthopaedics & trauma
Pregnancy & childbirth
Rheumatology
Skin
Tobacco, drugs, & alcohol dependence
Urology
Wounds

Box 5. Classification of journals as CAM or conventional

We will classify journals as having a CAM or conventional focus. We will initially classify journals as CAM if they met one or more of the following criteria, otherwise we will initially classify journals as having a conventional focus:

1) The journal is a Traditional Chinese Medicine (TCM) journal ;
2) The journal is in the subset of journals retrieved by the CAM on PubMed search strategy (http://www.nlm.nih.gov/bsd/pubmed_subsets/comp_med_strategy.html);
3) The journal is indexed with the broad subject of “Complementary therapies” in the NLM Catalog;
4) The journal is classified as “Integrative & Complementary Medicine” in Journal Citation Reports; or
5) More than 50% of MEDLINE articles in the journal are retrieved by the CAM on PubMed search strategy.

After initial classification of the top 50 CAM and top 50 conventional journals, we will have two authors independently classify the top 50 CAM and top 50 conventional journals as CAM or conventional in focus. Disagreement will be resolved by discussion. If journals change classification based upon this procedure, we will adjust the lists of top 50 CAM and top 50 conventional journals and repeat independent review of journal focus for each journal newly added to the group of top journals.
