# Supplementary material for: Bibliometric and content analysis of the Cochrane Complementary Medicine Field specialized register of controlled trials
Source: Syst Rev. 2013 Jul 4;2:51. doi: 10.1186/2046-4053-2-51 (PMC3704678; doi:10.1186/2046-4053-2-51)
Supplement: Additional file 2 — List of therapies included as CAM. [file 2046-4053-2-51-S2.doc]

**Additional file 2** **List of therapies included as CAM**

| Acupressure |
| --- |
| Acupuncture (e.g., needle acupuncture, electroacupuncture) |
| Alexander technique |
| Aromatherapy |
| Arts therapy (e.g., dance therapy, drama therapy, music therapy) |
| Ayurvedic traditional medicine (Ayurveda) |
| Balneotherapy |
| Bee products (e.g., honey, pollen, propolis, royal jelly, venom) |
| Biofeedback |
| Chelation therapy† |
| Chinese traditional medicine |
| Chiropractic (i.e., spinal manipulation) |
| Color therapy (i.e., chromotherapy) |
| Craniosacral manipulation |
| Dietary supplements (non-herbal) †(e.g., vitamins, hormones, amino acids) |
| Diet therapy† (e.g., low fat diets, vegan diets) |
| Distant healing |
| Electric stimulation therapy† (e.g., transcutaneous electrical nerve stimulation) |
| Electromagnetic therapy† |
| Eye Movement Desensitization and Reprocessing (EMDR) |
| Feldenkrais method |
| Herbal supplements (e.g., echinacea, garlic) |
| Homeopathy |
| Hydrotherapy |
| Hyperbaric oxygenation† |
| Hypnosis |
| Imagery (i.e., visualization techniques) |
| Light therapy† (phototherapy) |
| Magnetic field therapy† (e.g., transcranial magnetic stimulation) |
| Massage |
| Meditation |
| Morita therapy |
| Moxibustion |
| Naturopathy |
| Osteopathic manipulation |
| Ozone therapy† |
| Play therapy |
| Prolotherapy |
| Qi gong |
| Reflexology |
| Reiki therapy |
| Relaxation techniques |
| Snoezelen |
| Speleotherapy |
| Spiritual healing (e.g., prayer) |
| Tai chi |
| Therapeutic touch |
| Traditional healers and healing practices (other than Chinese) (e.g., Kampo, Shamanism) |
| Tui na |
| Ultrasonic therapy† |
| Yoga |

†Depending upon the condition being treated, these therapies may also be standard Western allopathic treatments. (Reproduced with permission from Wieland LS*, Manheimer E*, Berman BM: **Development and classification of an operational definition of complementary and alternative medicine for the Cochrane collaboration.** *Altern Ther Health Med* 2011, **17:**50**–**59.)
