# Supplementary material for: Bibliometric and content analysis of the Cochrane Complementary Medicine Field specialized register of controlled trials
Source: Syst Rev. 2013 Jul 4;2:51. doi: 10.1186/2046-4053-2-51 (PMC3704678; doi:10.1186/2046-4053-2-51)
Supplement: Additional file 3 — CAM search strategies for CAM interventions. [file 2046-4053-2-51-S3.pdf]

Acupuncture	
	1 exp acupuncture therapy/ or acupuncture therapy.tw. or (exp acupuncture analgesia/ or acupuncture analgesia.tw.) or (exp acupuncture points/ or acupuncture points.tw.) or (exp "acupuncture, ear"/ or ear acupuncture.tw.) or (exp acupressure/ or acupressure.tw.) or (exp acupuncture/ or acupuncture therapy.ti,ab. or exp acupuncture therapy/ or acupuncture.tw.) or (exp electroacupuncture/ or electroacupuncture.tw.) or auriculoacupuncture.mp. or dry needling.mp. or trigger point injection.mp. or trigger point injections.mp. or yoruba.mp. or neiguan.mp. or atuo.mp. or (Intramuscular adj3 Stimulation).mp.<br>2 limit 1 to (controlled clinical trial or randomized controlled trial) (2568)<br><br>Registry:<br>acupuncture or 'acupuncture therapy' or 'acupuncture analgesia' or 'acupuncture points' or 'ear acupuncture' or acupressure or electroacupuncture or auriculoacupuncture or 'dry needling' or 'trigger point injection' or yoruba or neiguan or atuo or 'intramuscular stimulation'<br>Yield=6035<br><br>Notes: The definition used for acupunction was this:<br>“The term “acupuncture” describes a family of procedures involving the stimulation of anatomical points on the body using a variety of techniques. The acupuncture technique that has been most often studied scientifically involves penetrating the skin with thin, solid, metallic needles that are manipulated by the hands or by electrical stimulation.” Electroacupunture, dry needling and acupressure are put here.<br><br>National Centre For Complementary and Alternative Medicine. Acupuncture: An Introduction [Internet]. NCCAM Pub No.: D404. 2007 [cited 2012 Apr 7];Available from: http://nccam.nih.gov/health/acupuncture/introduction.htm
**Chinese herbal medicine**	
	Medicine, Chinese Traditional/ or Drugs, Chinese Herbal/ OR Moxibustion/<br><br>OR<br><br>chinese medicine.tw. or chinese traditional medicine.tw. or Chinese herbal medicine.tw. OR Chinese herbal therapy.tw. or fu zheng.tw. or (huangdi and neijing).tw. or moxibustion.tw. or acumoxa.tw. OR Chinese Patent Medicine.tw. OR Chinese Herbal Extract*.tw. OR TCM.tw. OR Chinese herbology.tw.<br><br>***OR***<br><br>(Amur cork tree OR Baikal Skullcap OR Blue Evergreen Hydrangea OR Chinese Quinine OR Cassia OR Chinese Cinnamon OR Chaulmoogra tree OR Chinese Alangium Root OR Chinese anemone OR Chinese Arborvitae OR Chinese astragalus OR Chinese Cucumber OR Chinese ephedra OR Chinese Foxglove OR Chinese Goldthread OR Chinese Honeylocust OR Chinese Magnolia Vine OR Chinese motherwort OR Chinese rhubarb or Eastern rhubarb OR Costus OR Creeping Lobelia OR Devil's Trumpet OR Fumewort OR Golden Larch OR Hardy rubber tree OR Indian stringbush OR Jimson Weed OR Korean Mint OR Kudzu OR Licorice OR Lilac Dahpne OR Marlberry OR Naked rue OR Noble Dendrobium OR Pagoda Tree OR Peking spurge OR Purging Croton OR Purple Holly OR Safflower OR Sarpagandha OR Indian Snakeroot OR Stephania Root OR Szechuan lovage OR Tatar aster OR

Tartar aster OR Tea Plant OR Velvet leaf OR Weeping Forsythia).mp.

OR

(Agastache rugosa OR Alangium chinense OR Anemone chinensis OR Pulsatilla chinensis OR Anisodus tanguticus OR Ardisia japonica OR Aster tataricus OR Astragalus propinquus OR Astragalus membranaceus OR Camellia sinensis OR Carthamus tinctorius OR Cinnamomum cassia OR Cissampelos pareira OR Coptis chinensis OR Corydalis ambigua OR Croton tiglium OR Daphne genkwa OR Datura metel OR Datura stramonium OR Datura tatula OR Dendrobium nobile OR Dichroa febrifuga OR Ephedra sinica OR Eucommia ulmoides OR Euphorbia pekinensis OR Flueggea suffruticosa OR Securinega suffruticosa OR Forsythia suspensa OR Gentiana loureiroi OR Gleditsia sinensis OR Glycyrrhiza uralensis OR Hydnocarpus anthelminticus OR anthelminthica OR Ilex purpurea OR Leonurus japonicus OR Ligusticum wallichii OR Lobelia chinensis OR Phellodendron amurense OR Platycladus orientalis OR Thuja orientalis OR Pseudolarix amabilis OR Psilopeganum sinense OR Pueraria lobata OR Rauwolfia serpentina OR Rehmannia glutinosa OR Rheum officinale OR Rhododendron tsinghaiense OR Saussurea costus OR Schisandra chinensis OR Scutellaria baicalensis OR Stemona tuberosa OR Stephania tetrandra OR Styphnolobium japonicum OR Sophora japonica OR Trichosanthes kirilowii OR Wikstroemia indica).mp.

Yields 2796 RCT or CCTs in MEDLINE
the first 2 lines Yield 2554 of these

Registry (This search needs to be run in blocks of <1028 characters)

#45='Medicine, Chinese Traditional' OR #45=' Drugs, Chinese Herbal' OR 'chinese medicine' or 'chinese traditional medicine' or 'Chinese herbal medicine' OR 'Chinese herbal therapy' or 'fu zheng' or (huangdi and neijing) or moxibustion or acumoxa OR 'Chinese Patent Medicine' OR 'Chinese Herbal Extract*' OR TCM OR 'Chinese herbology'

OR

'Amur cork tree' OR 'Baikal Skullcap' OR 'Blue Evergreen Hydrangea' OR 'Chinese Quinine' OR Cassia OR 'Chinese Cinnamon' OR 'Chaulmoogra tree' OR 'Chinese Alangium Root' OR 'Chinese anemone' OR 'Chinese Arborvitae' OR 'Chinese astragalus' OR 'Chinese Cucumber' OR 'Chinese ephedra' OR 'Chinese Foxglove' OR 'Chinese Goldthread' OR 'Chinese Honeylocust' OR 'Chinese Magnolia Vine' OR 'Chinese motherwort' OR 'Chinese rhubarb' or 'Eastern rhubarb' OR Costus OR 'Creeping Lobelia' OR 'Devil* Trumpet' OR Fumewort OR 'Golden Larch' OR 'Hardy rubber tree' OR 'Indian stringbush' OR 'Jimson Weed' OR 'Korean Mint' OR Kudzu OR Licorice OR 'Lilac Dahpne' OR Marlberry OR 'Naked rue' OR 'Noble Dendrobium' OR 'Pagoda Tree' OR 'Peking spurge' OR 'Purging Croton' OR 'Purple Holly' OR Safflower OR Sarpagandha OR 'Indian Snakeroot' OR 'Stephania Root' OR 'Szechuan lovage' OR 'Tatar aster' OR 'Tea Plant' OR 'Velvet leaf' OR 'Weeping Forsythia'

OR
'Agastache rugosa' OR 'Alangium chinense' OR 'Anemone chinensis' OR 'Pulsatilla chinensis' OR 'Anisodus tanguticus' OR 'Ardisia japonica' OR 'Aster tataricus' OR 'Astragalus propinquus' OR 'Astragalus membranaceus' OR 'Camellia sinensis' OR 'Carthamus tinctorius' OR 'Cinnamomum cassia' OR 'Cissampelos pareira' OR 'Coptis

	chinensis' OR 'Corydalis ambigua' OR 'Croton tiglium' OR 'Daphne genkwa' OR 'Datura metel' OR 'Datura stramonium' OR 'Datura tatula' OR 'Dendrobium nobile' OR 'Dichroa febrifuga' OR 'Ephedra sinica' OR 'Eucommia ulmoides' OR 'Euphorbia pekinensis' OR 'Flueggea suffruticosa' OR 'Securinega suffruticosa'<br><br>OR<br>'Forsythia suspensa' OR 'Gentiana loureiroi' OR 'Gleditsia sinensis' OR 'Glycyrrhiza uralensis' OR 'Hydnocarpus anthelminticus' OR anthelminthica OR 'Ilex purpurea' OR 'Leonurus japonicus' OR 'Ligusticum wallichii' OR 'Lobelia chinensis' OR 'Phellodendron amurense' OR 'Platycladus orientalis' OR 'Thuja orientalis' OR 'Pseudolarix amabilis' OR 'Psilopeganum sinense' OR 'Pueraria lobata' OR 'Rauwolfia serpentina' OR 'Rehmannia glutinosa' OR 'Rheum officinale' OR 'Rhododendron tsinghaiense' OR 'Saussurea costus' OR 'Schisandra chinensis' OR 'Scutellaria baicalensis' OR 'Stemona tuberosa' OR 'Stephania tetrandra' OR 'Styphnolobium japonicum' OR 'Sophora japonica' OR 'Trichosanthes kirilowii' OR 'Wikstroemia indica' OR cannabis OR marijuana<br><br>Yield = 12,118<br><br>Notes:<br>The TCM search is based on the PubMed CAM strategy supplemented with the common and scientific names of 50 of the fundamental herbs. As well, we examined the searches for two systematic reviews identified by Hu *et al.* as including a broad base of TCM. Acupuncture is not included here, as it has its own topic, however acumoxa, which is a combination of acupuncture & moxibustion, is placed only here, not also in with the acupuncture topic search. Certain other TCM practices that are not unique to TC but are used in a variety of traditions are covered elsewhere, include cupping, massage, mind-body therapy, and dietary therapy. Cannabis appears in the PubMed CAM strategy but it was removed from the MEDLINE search after testing – it accounted for almost 15000 hits in MEDLINE and none were indexed as TCM. It was retained in the Registry search.<br><br>Additional sources consulted:<br>Chinese Herbology, 50 Fundamental Herbs. Wikipedia. Available at http://en.wikipedia.org/wiki/Chinese_herbology<br><br>Liu JP, Zhang M, Wang WY, Grimsgaard S. Chinese herbal medicines for type 2 diabetes mellitus. Cochrane Database Syst Rev. 2004 Jan;(3):CD003642.<br><br>Zhu X, Proctor M, Bensoussan A, Wu E, Smith CA. Chinese herbal medicine for primary dysmenorrhoea. Cochrane Database of Systematic Reviews. 2010.<br><br>These 2 systematic reviews were chosen from Hu *et al.* because they covered large numbers of TCM herbs. One other (Tao Gan) included more than Zhu, but had been withdrawn as out of date.<br><br>Hu J, Zhang J, Zhao W, Zhang Y, Zhang L, Shang H. Cochrane systematic reviews of Chinese herbal medicines: an overview. PloS one. 2011 Jan;6(12):e28696.
**Homeopathy**	
	Homeopathy/ OR Formularies, Homeopathic/ OR Pharmacopoeias, Homeopathic/

	OR Materia Medica/<br>OR<br>(homeopath$ OR homoeopath* OR homotoxi* OR materia medica OR pharmacopoeia* OR single remedy OR combination remed* OR infinitesimals OR similars isopath* OR traumeel).mp.<br><br>MEDLINE = 252 CAM RCT or CCT<br><br><br>Registry:<br>homeopath* OR homoeopath* OR homotoxi* OR 'materia medica*' OR formular* OR pharmacopoeia* OR 'single remedy' OR 'combination remed*' OR infinitesimals OR similars OR isopath* OR traumeel<br><br>Yield= 755<br><br>Additional sources consulted:<br>Milazzo S, Russell N, Ernst E. Efficacy of homeopathic therapy in cancer treatment. Eur. J. Cancer. 2006 Feb;42(3):282-9.
**Traditional medicine not otherwise specified** (e.g., Ayurveda, Kampo)	
	Medicine, Tradtional/ OR Medicine, African Traditional/ OR Medicine, Arabic/ OR Medicine, Unani/ OR Medicine, Ayurvedic/ OR Medicine, East Asian Traditional/ OR Medicine, Kampo/ OR Medicine, Korean Traditional/ OR Medicine, Mongolian Traditional/ OR Shamanism/ OR ((north american indians.tw. or indians, north american/ or native american.tw.) and (medicine, traditional/ or traditional medicine.tw.))<br>or<br>unani.mp. or tibbi.tw. or tibb.tw. or african traditional medicine.tw. or traditional african medicine.tw. or curanderismo.mp. or (tibetan.mp. and pharmaceutical.mp.) or (Tibetan.mp. and medicine.mp.) or kampo.tw. or ((aboriginal or aborigines).mp. and (medicinal.mp. or pharmaceutical.mp. or medicine.mp.)) or arabic medicine.tw. or la'au.mp. or somoan herbal medicine.mp. or somoan medicine.mp. or yoruba.mp.<br>or<br>traditional medicine.tw. or primitive medicine.tw. or folk medicine.tw. or folklore/ or folklore.tw. or folk.tw. or (traditional and indigenous).mp. or home remedies.tw. or home remedy.tw. or traditional healer*.mp.<br>or<br>(ancient Iranian medicine OR Islamic medicine OR Indigenous medicine OR kanpo OR Muti OR Shaman* OR traditional Korean medicine).tw.<br>or<br>(Ayurved* Or panchakarma or Devanagari or rasayana or Vajikaranam or Kaya-cikitsa or Kaumarabhrtyam or Salya-cikitsa or Salakya tantra or Bhuta or Agadatantram Or unmade).mp.<br>or<br>(adhatoda vasica or albizzia lebbeck or andrographis paniculata or bacopa monniera or coleus forskohlii or commiphora mukul or crataeva nurvala or gymnema sylvestre or hemidesmus indicus or inula racemosa or phyllanthus amarus or picrorrhiza kurroa or terminalia arjuna or tylophora indica or withania somnifera).mp.<br>or

(fenugreek or foenum* or momordica charantia or cinnamomum tamala or pterocarpus marsupium or eugenia jambolina or Trigonella OR coccinea indica or pancreas tonic*).mp.

= 346 RCT or CCT in MEDLINE
137 trials for aruvedic (last 3 chunks)

Registry (This search needs to be run in blocks of <1028 characters)

#45='Medicine, Tradtional' OR #45='Medicine, African Traditional' OR #45='Medicine, Arabic' OR #45='Medicine, Unani' OR #45='Medicine, Ayurvedic' OR #45='Medicine, East Asian Traditional' OR #45='Medicine, Kampo' OR #45='Medicine, Korean Traditional' OR #45='Medicine, Mongolian Traditional' OR (('north american indians' or #45='Indians, north american' OR 'native american') AND 'traditional medicine') OR unani OR tibbi OR tibb OR 'african traditional medicine' OR 'traditional african medicine' OR curanderismo OR (Tibetan AND pharmaceutical) OR (Tibetan AND medicine) OR kampo OR ((aboriginal OR aborigines) AND (medicinal OR pharmaceutical OR medicine)) OR 'arabic medicine' OR 'somoan herbal medicine' OR 'somoan medicine' OR yoruba OR 'traditional medicine' OR 'primitive medicine' OR 'folk medicine' OR folklore OR folk OR (traditional AND indigenous) or 'home remed*' OR 'traditional healer*'

'ancient Iranian medicine' OR 'Islamic medicine' OR 'Indigenous medicine' OR Muti OR Shaman* OR 'traditional Korean medicine' OR Ayurved* Or panchakarma OR Devanagari OR rasayana OR Vajikaranam OR 'Kaya-cikitsa' OR Kaumarabhrtyam OR 'Salya-cikitsa' OR 'Salakya tantra' OR Bhuta OR Agadatantram OR unmade OR 'adhatoda vasica' OR 'albizzia lebbeck' OR 'andrographis paniculata' OR 'bacopa monniera' OR 'coleus forskohlii' OR 'commiphora mukul' OR 'crataeva nurvala' OR 'gymnema sylvestre' OR 'hemidesmus indicus' OR 'inula racemosa' OR 'phyllanthus amarus' OR 'picrorrhiza kurroa' OR 'terminalia arjuna' OR 'tylophora indica' OR 'withania somnifera' OR fenugreek OR foenum* OR 'momordica charantia' OR 'cinnamomum tamala' OR 'pterocarpus marsupium' OR 'eugenia jambolina' OR Trigonella OR 'coccinea indica' OR 'pancreas tonic*'
total =1409

Additonal sources consulted:
Hardy M, Coulter I, Venuturupalli S, et al. Ayurvedic Interventions for Diabetes Mellitus: A Systematic Review. Evidence Reports/Technology Assessments, No. 41. Rockville (MD): Agency for Healthcare Research and Quality (US); 2001 Sep. http://www.ncbi.nlm.nih.gov/books/NBK33788/ PMID 11488136

Sridharan K, Mohan R, Ramaratnam S, Panneerselvam D. Ayurvedic treatments for diabetes mellitus. Cochrane Database Syst Rev. 2011 Dec 7;12:CD008288. Review. PubMed PMID: 22161426.

Agarwal V, Abhijnha A, Raviraj P. Ayurvedic medicine for schizophrenia. Cochrane Schizophrenia Group Cochrane Database of Systematic Reviews. 3, 2010.

Chelation therapy

	1 Chelating Agents/ or Iron Chelating Agents/ or Siderophores/ or Chelation Therapy/ or chelat*.tw. 54802<br>2 limit 1 to (controlled clinical trial or randomized controlled trial) 568<br>3 limit 2 to complementary medicine 292<br>4 3 not (feric or ferrous or iron or thalassemia).mp. 190<br><br>**Registry strategy (specific):**<br>Chelating Agent*' or 'Iron Chelating Agent*' or 'Siderophores' or 'Chelation Therapy' or chelat*<br>Yields = 134
**Diet-based therapies** (e.g., vegetarian diets) Any Diet = 9009	
	1 Diet/ (95598)<br>2 Diet Therapy/ (9119)<br>3 (food adj2 choice$).tw. (2129)<br>4 Nutrition Therapy/ (689)<br>5 Nutritional Support/ (4231)<br>6 OR/1-5 (110705)<br>7 limit 6 to (controlled clinical trial or randomized controlled trial) (5322)<br><br>Registry<br>Diet OR 'food choice*' OR nutrition<br>Yield = 7510
Ketogenic Diet	
	1 Ketogenic Diet/ (275)<br>2 (ketogenic diet or ketogenic dieting or ketogenic diets).tw. (996)<br>3 Ketone Bodies/ (4635)<br>4 Ketosis/ (1234)<br>5 Ketones/ (10697)<br>6 OR/1-5 (16830)<br>7 limit 6 to (controlled clinical trial OR randomized controlled trial) (274)<br><br>Registry:<br>keytone OR ketogenic OR ketosis<br>Yield=44
Low calorie Diet	
	1 Diet, Reducing/ (8721)<br>2 Caloric Restriction/ (2799)<br>3 (low adj2 calorie).tw. (1987)<br>4 (calorie adj2 reduc$).tw. (418)<br>5 or/1-4 (12637)<br>6 limit 5 to (controlled clinical trial or randomized controlled trial) (1683)<br>7 limit 6 to complementary medicine (383)<br><br>Registry:

	'low cal*' or 'calori* restrict*' or 'calori* reduc*' or 'reduced calori*' or 'reduc* diet'<br>Yield=87
Low bacterial diet	
	1 (neutropenic adj3 food).tw. (0)<br>2 (neutropenic adj3 diet).tw. (8)<br>3 (reduc$ adj3 bacteria$).tw. (8797)<br>4 (low adj3 fung$).tw. (323)<br>5 (reduc$ adj3 fung$).tw. (1046)<br>6 (low adj2 bacteria$).tw. (838)<br>7 Diet/ (95598)<br>8 Diet Therapy/ (9119)<br>9 (Diet* adj3 therapy).tw. (4335)<br>10 Food/ (21282)<br>11 diet*.tw. (336562)<br>12 eat*.tw. (50798)<br>13 OR/3-6 (10934)<br>14 OR/7-12 (429995)<br>15 13 and 14 (318)<br>16 1 OR 2 (8)<br>17 15 OR 16 (326)<br>18 limit 17 to (controlled clinical trial OR randomized controlled trial) (25)<br><br>Registry:<br>neutropen* diet' OR 'neutropen* food' OR 'low bacteri*' OR 'reduced bacteria*' OR 'low fung*' OR 'reduc* fung*'<br>Yield=2
Vegetarian or Vegan diet	
	1 Diet, Vegetarian/ (2348)<br>2 vegetar*.tw. (2421)<br>3 vegan*.tw. (362)<br>4 (vegetarian adj3 diet).tw. (735)<br>5 (vegetarian adj3 food).tw. (78)<br>6 Diet, Macrobiotic/ (46)<br>7 Macrobiotic.tw. (89)<br>8 OR/1-7 (3400)<br>9 limit 8 to (controlled clinical trial OR randomized controlled trial) (192)<br><br>Registry:<br>vegetar* OR vegan* OR 'vegetarian diet' OR macrobiotic<br>Yield=187
Gluten-free diet	
	1 Diet, Gluten-Free/ (440)<br>2 (gluten-free OR gluten free).tw. (2860)<br>3 Glutens/ (4353)<br>4 OR/1-3 (6150)<br>5 limit 4 to (controlled clinical trial OR randomized controlled trial) (110)<br>6 limit 5 to complementary medicine (17)

	Registry:<br>gluten OR 'gluten-free' OR 'gluten free'<br>Yield=20
Fasting	
	1 Fasting/ (26325)<br>2 (master adj3 cleanse).tw. (0)<br>3 (detox adj2 diet).tw. (0)<br>4 (detoxification adj2 diet).tw. (4)<br>5 OR/1-4 (26329)<br>6 limit 5 to (controlled clinical trial OR randomized controlled trial) (1966)<br><br>Registry:<br>fast OR fasting OR cleanse OR 'master cleanse' OR detox* OR de-tox*<br>Yield=1647
Mediterranean diet	
	1 Diet, Mediterranean/ (1077)<br>2 (mediterranean adj2 diet).tw. (1475)<br>3 (mediterranean adj2 food).tw. (75)<br>4 dash diet.tw. (151)<br>5 OR/1-4 (1961)<br>6 limit 5 to (controlled clinical trial OR randomized controlled trial) (240)<br><br>Registry:<br>Mediterranean OR 'dash diet'<br>Yield=207
Macrobiotic diet	
	See vegetarianism
Paleolithic diet	
	1 Paleolithic diet.mp. (22)<br>2 (caveman or (cave adj1 man)).tw. (104)<br>3 hunter-gatherer.mp. (295)<br>4 or/1-3 (416)<br>5 limit 4 to (controlled clinical trial or randomized controlled trial) (3)<br><br>Registry:<br>Paleolithic OR caveman OR 'stone age' OR hunter-gatherer<br>Yield=2
Low salt diet	

	1 Diet, Sodium-Restricted/ (5178)<br>2 low sodium.tw. (2579)<br>3 (salt adj3 reduc*).tw. (1821)<br>4 low salt.tw. (3803)<br>5 dash diet.tw. (151)<br>6 OR/1-5 (11755)<br>7 limit 6 to (controlled clinical trial OR randomized controlled trial) (731)<br>8 limit 7 to complementary medicine (81)<br><br>Registry:<br>low salt' OR 'sodium restrict*' OR 'sodium-restrict*' OR 'low sodium' OR 'dash diet' OR 'sodium reduc*' OR 'sodium-reduc*' OR 'salt restrict*' OR 'salt reduc*'<br>Yield=71
Functional Food diet	
	1 Functional Food/ (310)<br>2 Food, Fortified/ (6985)<br>3 Prebiotics/ (355)<br>4 nutraceutical.tw. (55)<br>5 Micronutrients/ (2599)<br>6 Probiotics/ (6723)<br>7 Synbiotics/ (47)<br>8 Yeast, Dried/ (694)<br>9 functional food*.tw. (1892)<br>10 Health Food/ (1420)<br>11 or/1-10 (19831)<br>12 limit 11 to (controlled clinical trial or randomized controlled trial) (2358)<br><br>Registry:<br>'functional food' or fortified or prebiotic or nutraceutical or micronutrient or probiotic or synbiotic or yeast or 'health food'<br>Yield=1061
Low carbohydrate diet	
	1 Diet, Carbohydrate-Restricted/ (579)<br>2 low carb*.tw. (2040)<br>3 atkin$ diet.tw. (121)<br>4 (south adj2 beach adj2 diet).tw. (8)<br>5 (dukan adj2 diet).tw. (0)<br>6 (low adj2 carb*).tw. (3480)<br>7 (carbohydrate adj2 restrict*).tw. (331)<br>8 OR/1-7 (4036)<br>9 limit 6 to (controlled clinical trial OR randomized controlled trial) (387)<br><br>REGISTRY:<br>Carbohydrate-Restricted OR 'low-carbohydrate' OR 'carb* restrict*' OR 'low carb*' OR atkins OR 'south beach' OR dukan<br>Yield=238
Low fat diet	
	1 Diet, Fat-Restricted/ (2399)

	2 (low adj3 fat).tw. (9205)<br>3 Dietary Fats, Unsaturated/ (4323)<br>4 (fat adj2 restrict*).tw. (440)<br>5 (restrict* adj2 fat*).tw. (683)<br>6 OR/1-5 (14991)<br>7 limit 6 to (controlled clinical trial OR randomized controlled trial) (2163)<br>8 limit 7 to complementary medicine (1170)<br><br>Registry:<br>'low fat' OR 'restrict* fat' OR 'fat free' OR 'fat restrict*' OR 'dietary fat'<br>Yield=304
Elimination diet	
	1 Food Hypersensitivity/ (11038)<br>2 (hypoallergenic adj2 diet).tw. (56)<br>3 (elimination adj2 diet).tw. (424)<br>4 (elemental adj2 diet).tw. (733)<br>5 food allergy.tw. (4334)<br>6 egg hypersensitivity/ OR milk hypersensitivity/ OR nut hypersensitivity/ OR peanut hypersensitivity/ OR wheat hypersensitivity/ (2458)<br>7 OR/1-6 (14802)<br>8 limit 7 to (controlled clinical trial OR randomized controlled trial) (637)<br><br>Registry:<br>'elimination diet' OR 'food allergy' OR hypoallergenic OR 'elemental diet' OR 'food hypersensitivity' OR wheat OR peanut OR nut OR milk OR dairy<br>Yield=1219
**Non-vitamin, non-mineral dietary supplements and herbal products** (e.g., probiotics, ginseng)	
	1 Plants, Medicinal/ or Dietary Supplements/ or Prebiotics/ or Probiotics/ or Synbiotics/ or Orthomolecular therapy/ or Oils, Volatile/tu or Aromatherapy/ (80103)<br>2 (botanical medicine or herbal* or herbal medicine or herbology or herblore or cellasene or aromatherapy or essential oil* or royal jelly or dietary supplement* or micronutrient* or orthomolecular or taurine or caffeine or caffeic acid or coenzyme q10 or coq10 or melatonin or echinacea or valerian or yohimbine or yohimbe or allium sativum or garlic or ginkgo or ginkgolides or panax or ginseng or acai or glucosamine or chondroitin or (mushroom* and (medicinal or therapy or antifungal or antineoplastic)) or (proteolytic enzymes and cancer) or linoleic acid or (dietary fats and unsaturated fatty acids) or omega-3 or fish oil or ((n3 or n-3) and (oil or oils or puFa or fatty acid or fatty acids)) or antioxidant* or flavonoid* or flavinoid* or bioflavinoid* or bioflavonoid* or flavonol* or flavone* or flavanone* or isoflavone* or catechin* or anthocyanidin* or chalcone* or functional food* or antineoplaston* or (creatine and huntington*) or shark cartilage or powdered cartilage or thymus extract or pancreatic extract).mp. (344631)<br>3 (1 or 2) not (chinese or traditional or vitamin* or mineral*).mp. (314881)<br>4 limit 3 to (controlled clinical trial or randomized controlled trial) (12847)<br>5 limit 4 to complementary medicine (7769)<br><br>Phytotherapy OR phytomedicine OR #45='Plants, Medicinal' OR Prebiotic* OR Probiotic* OR Synbiotic* OR Orthomolecular OR #45='Oils, Volatile' OR Aromatherapy OR botanical OR herb* OR cellasene OR 'essential oil' OR 'royal jelly' OR 'dietary supplement*' OR micronutrient* OR taurine OR caffe* OR 'coenzyme q10'

	OR coq10 OR melatonin OR echinacea OR valerian OR yohimbine OR yohimbe OR 'allium sativum' OR garlic OR ginkgo* OR panax OR ginseng OR acai OR glucosamine OR chondroitin OR mushroom* OR antifungal OR antineoplastic OR proteolytic OR 'linoleic acid' OR 'omega 3' OR 'fish oil' OR pufa or 'fatty acid*' OR antioxidant* OR flavonoid* OR flavinoid* OR bioflav* OR flavon* OR isoflavone* OR catechin* OR anthocyanidin* OR chalcone* OR 'functional food*' OR antineoplaston* OR 'shark cartilage' OR cartilage OR 'thymus extract' or 'pancreatic extract' OR extract<br>Yield=15,140<br>Note: This category covers, roughly, things ingested, not otherwise specified. This category is difficult to delineate precisely from TCM, other traditional medicines, vitamins and minerals. It must be considered approximate and to be used only with the registry. Readers are advised to consult the Search Strategy Used to Create the Dietary Supplements Subset on PubMed for a more complete treatment of this topic.<br><br>1. National Libary of Medicine, Office of Dietary Supplements. Search Strategy Used to Create the Dietary Supplements Subset on PubMed [Internet]. 2011 [cited 2012 Mar 15];Available from: http://www.nlm.nih.gov/bsd/pubmed_subsets/diet_strategy.htm
**Vitamin and mineral therapies** (includes megavitamin therapies and vitamin or mineral therapies for other than medically diagnosed deficiencies or deficiency-related disorders)	
	1 Vitamins/ (16461)<br>2 vitamin.mp. (162785)<br>3 Minerals/ (15772)<br>4 mineral*.mp. (107350)<br>5 Androstenedione/ (6233)<br>6 androstenedione.mp. (10108)<br>7 andro.mp. (101)<br>8 Ascorbic Acid/ (33105)<br>9 vitamin c.mp. (13639)<br>10 Biotin/ (11155)<br>11 Boron/ (2389)<br>12 Bromelains/ (1083)<br>13 Calcium/ (229675)<br>14 Carotenoids/ (12922)<br>15 Choline/ (14634)<br>16 Chromium/ (9469)<br>17 Colloids/ or Silver/ or colloidal silver.mp. (23032)<br>18 Linoleic Acids, Conjugated/ (1309)<br>19 Copper/ (47655)<br>20 Docosahexaenoic Acids/ (4731)<br>21 Folic Acid/ (18724)<br>22 dha.mp. (7019)<br>23 Inositol/ (6196)<br>24 Iodine/ (17001)<br>25 Iron/ (69200)<br>26 l-arginine.mp. or Arginine/ (53464)<br>27 Magnesium/ (59399)

	28 Manganese/ (19327)<br>29 Molybdenum/ (5578)<br>30 Niacin/ (5178)<br>31 Niacinamide/ (6008)<br>32 Phosphatidylcholines/ (27090)<br>33 Lecithins/ (316)<br>34 Potassium/ (91571)<br>35 Pyridoxine/ (6654)<br>36 exp Riboflavin/ (10456)<br>37 selenium.mp. or Selenium/ (23284)<br>38 retinoid.mp. or Retinoids/ (13733)<br>39 Silicon/ (7380)<br>40 thiamine.mp. or Thiamine/ (12832)<br>41 Tocopherols/ (1850)<br>42 Vanadium/ or vanadium.mp. (5873)<br>43 vitamin a.mp. or Vitamin A/ (26696)<br>44 Vitamin D/ or vitamin d.mp. (42758)<br>45 Vitamin E/ or vitamin e.mp. (30319)<br>46 vitamin k.mp. (14029)<br>47 Yeast, dried/ or yeast.mp. (127005)<br>48 Zinc/ or zinc.mp. (93970)<br>49 or/1-48 (1141487)<br>50 limit 49 to (controlled clinical trial or randomized controlled trial) (23874)<br>51 limit 50 to complementary medicine (6795)<br><br>Registry:<br>Vitamin* OR mineral* OR yeast OR androstenedione OR andro OR 'arachidonic acid' OR 'ascorbic acid' OR 'vitamin c' OR biotin OR boron OR bromelain* OR calcium OR caroten* OR choline OR chromium OR copper OR 'colloidal silver' OR 'conjugated linoleic acid' OR 'docosahexaenoic acid' OR dha OR 'folic acid' OR inositol OR iodine OR iron OR 'l arginine' OR magnesium OR manganese OR 'molybdenum 4' OR niacin* OR 'phosphatidyl choline' OR lecithin OR potassium OR pyridoxine OR riboflavin OR retinoid* OR retinoic OR retinal OR selenium OR silicon OR thiamine OR tocopherol* OR vanadium OR 'vitamin a' OR 'vitamin d' OR 'vitamin e' OR 'vitamin k' OR yeast OR zinc OR micronutrient*<br><br>Yield = 7,741<br><br>Notes:<br>This search is adapted from the Complementary Medicine Subset on PubMed.<br><br>All records in the CAM registry are assumed to represent CAM interventions, therefore. When this search is used in MEDLINE, it would be necessary for the user to determine if the the intervention could be considered CAM. This seach does not attempt to make that differentiation.
**Biologically based therapies not otherwise specified and excluding therapies using energy fields**<br>(e.g., balneotherapy	

Balneology/ OR Ammotherapy/ OR Health Resorts/ OR Mineral Waters/ OR Hydrotherapy/ OR Baths/ OR Mud Therapy/ OR Steam Bath/ OR Climatotherapy/ OR Hyperbaric Oxygenation/ NOT (wounds OR wound healing OR decompression sickness OR diving).mp. OR Ozone/tu OR Chronotherapy/ OR Mesotherapy/ OR Speleotherapy/ OR Hyperthermia, Induced/ or Cryotherapy/
OR
(bath OR baths OR bathing OR therapeutic irrigation OR balneopelotherap* OR spa OR balneotherap* OR balneolog* OR thalassother* OR climate ther* OR climatother* OR hydropath* OR hydrotherap* OR balneo-hydrother*
or sweat lodge OR ammotherap* OR mud OR sauna OR steam OR whirlpool OR whirl pool OR dead sea OR thermotherap* OR mud pack* OR mud therapy OR pelotherap* OR peloid therapy OR fangotherap* OR thalassotherapy OR ammotherap* OR aerotherapy OR oxygenotherapy OR ozone OR ozonotherapy OR oxymedicine OR hyperoxigenation OR altitude cure OR chronotherap* OR mesotherap* OR speleotherap* OR cold therap* OR cryotherap* OR apither* OR (maggot and therap*) OR heat therap* OR thermotherap* or fever therap* or induced hypertherm*).mp.
OR (hydrogen peroxide AND (intravenous OR infusion*) AND (dosage OR therap*)).mp. OR (hyperbaric oxygen* not (wounds or wound healing or decompression sickness or diving)).mp. OR (saline AND (nose OR nasal) AND irrigat*).mp. OR (maruyama AND vaccine*).mp.

MEDLINE = 1918

Registry
bath OR baths OR bathing OR 'therapeutic irrigation' OR balneopelotherap* OR spa OR 'health resort' OR balneotherap* OR balneolog* OR thalassother* OR climate OR climatother* OR hydropath* OR hydrotherap* OR balneo-hydrother* OR 'sweat lodge' OR ammotherap* OR 'mineral waters' OR mud OR sauna OR steam OR whirlpool* OR 'whirl pool' OR 'dead sea' OR thermotherap* OR mud OR pelotherap* OR 'peloid therapy' OR fangotherap* OR thalassotherapy OR ammotherap* OR aerotherapy OR oxygenotherapy OR ozone OR ozonotherapy OR oxymedicine OR hyperoxigenation OR 'hyperbaric oxygen' OR 'altitude cure' OR chronotherap* OR mesotherap* OR speleotherap* OR 'cold therapy' OR cryotherap* OR apither* OR 'maggot' OR 'heat therapy' OR thermotherap* or 'fever therapy' or hyperthermia OR 'hydrogen peroxide' OR 'hyperbaric oxygen*' OR 'maruyama vaccine*'

Yield = 1215

Note on the Procite translation: some terms, such as 'mud' and 'climate', that would ordinarily be modified with another term, such as 'therapy' were tested, and when they occurred infrequently in the database, the modifiers were dropped. For example, "mud" appears only three times in the registry. Therefore, 'mud' was searched alone, rather than 'mud therapy or mud therapies or therapeutic mud'.

Chiropractic or osteopathic manipulation

1 Manipulation, Chiropractic/ (635)
2 Chiropractic/ (2940)
3 Manipulation, Spinal/ (995)

	4 Manipulation, Orthopedic/ (3340)<br>5 Manipulation, Osteopathic/ (301)<br>6 Musculoskeletal Manipulations/ (705)<br>7 Osteopathic Medicine/ (2433)<br>8 (spin* adj3 (adjust* or manipul*)).tw. (1713)<br>9 (chiropract* or osteopath*).tw. (7342)<br>10 network spinal analysis.tw. (3)<br>11 pierce stillwagon.tw. (1)<br>12 torque release.tw. (3)<br>13 naprapathy.tw. (13)<br>14 Kinesiology, Applied/ (176)<br>15 applied kinesiology.tw. (57)<br>16 or/1-15 (14601)<br>17 limit 16 to (controlled clinical trial or randomized controlled trial) (791)<br><br>activator or 'atlas orthogonality' or biophysics or flexion or diversified or gonstead or hio or logan or manipulation or 'neural organiation' or 'neuro emotional' or sacro-occipital or thompson or toftness or toggle or 'upper cervical specific' or vax-d<br><br>Registry:<br>#45= Orthopedic or #45=Spinal or osteopath* or manipulat* or chiropract* or activator or adjustment or 'atlas orthogonality' or biophysics or flexion or diversified or gonstead or hio or kinesiology or logan or manipulation or 'network spinal analysis' or 'neural organiation' or 'neuro emotional' or 'pierce stillwagon' or sot or sacro-occipital or thompson or toftness or toggle or 'torque release' or 'upper cervical specific' or vax-d<br><br>Yield = 2,606<br><br>Additional sources consulted:<br>Walker BF, French SD, Grant W, Green S. Combined chiropractic interventions for low-back pain. Cochrane Database of Systematic Reviews 2010, Issue 4. Art. No.: CD005427. DOI: 10.1002/14651858.CD005427.pub2.<br><br>Rubinstein SM, van Middelkoop M, Assendelft WJJ, de Boer MR, van Tulder MW. Spinal manipulative therapy for chronic low-back pain. Cochrane Database of Systematic Reviews 2011, Issue 2. Art. No.: CD008112. DOI: 10.1002/14651858.CD008112.pub2.
**Massage**	
Medline	1 Massage/ (4278)<br>2 Reflexotherapy/ (365)<br>3 (tui and na).tw. (7)<br>4 (shiatsu or reflexology or reflexotherapy or Kneipp Cure or Jin Shin or hawaiian lomi or lomi lomi or effleurage or hellerwork).mp. (973)<br>5 (myofascial and release).mp. (141)<br>6 (trigger point therapy or amma).mp. (69)<br>7 (reflexotherapy or vagal-reflexotherapy or ultra-thoracic pressure or (valsalva adj2 maneuver)).mp. (4715)

	8 (reichian or petrissage or watsu or myotherapy).mp. (45)<br>9 (manual* and lymph* and drainage).mp. (251)<br>10 (meridian and therapy).mp. (326)<br>11 (neurocranial and restructuring).mp. (2)<br>12 (neuromuscular and integrated and action).mp. (45)<br>13 or/1-12 (9924)<br>14 limit 13 to (controlled clinical trial or randomized controlled trial) (820)<br><br>Registry:<br>Massage or 'Active Release Techniques' or 'Graston technique' or effleurage or massotherapy or petrissage or bodywork or 'body work' or watsu or shiatsu or acupressure or 'swedish massage' or 'thai massage' or 'deep tissue massage' or 'trigger point therapy' or 'reflexology'<br><br>Yield = 1,481<br><br>Notes:<br>Some of the terms could equally be placed with manipulation therapies.
**Manipulative and body based therapies not otherwise specified**<br>(e.g., Alexander technique, Pilates)	
	1 Pilates.mp. 83<br>2 Rolfing.mp. 17<br>3 Hellerwork.mp. 1<br>4 Structural integration.mp. 118<br>5 feldenkrais.mp. 44<br>6 (Functional Integration or Awareness Through Movement).mp. 695<br>7 Kinotherapy.mp. 0<br>8 bobath.ti,ab. 157<br>9 (neuro-developmental treatment or neurodevelopmental treatment).mp. 67<br>10 Vibration/ or full body vibration.mp. 17569<br>11 Vibration/ or full body vibration.mp. 17569<br>12 bates method.mp. 6<br>13 Bloodletting/ 2393<br>14 (bloodletting or blood letting).tw. 681<br>15 (cupping or vacuum cup therapy or Hijama).mp. 1077<br>16 Alexander technique.mp. 54<br>17 (Bowen Technique or Bowen Therapy or Bowtech or Bowenwork).mp. 11<br>18 or/1-17 22591<br>19 limit 18 to (controlled clinical trial or randomized controlled trial) 774<br>20 limit 19 to complementary medicine 207<br><br>MEDLINE RCT CCT & CAM = 207<br><br>Registry:<br>vibration OR bloodletting OR 'blood letting' OR 'blood-letting' OR Pilates Or Rolfing OR Hellerwork OR 'structural integration' OR feldenkrais OR 'functional integration' OR 'awareness through movement' OR kinotherapy OR 'neuro-developmental

	treatment' OR 'neurodevelopmental treatment' or 'full body vibration' OR 'full-body vibration' OR 'bates method' OR cupping OR 'vacuum cup therapy' OR Hijama OR 'Alexander technique' OR 'Bowen Technique' or 'Bowen Therapy' or Bowtech or Bowenwork OR Bobath<br><br>Yield = 438<br><br>Additional sources consulted:<br>Hansen C, Taylor-Piliae RE. What is Bowenwork? A systematic review. J Altern Complement Med. 2011 Nov;17(11):1001-6.
**Biofeedback**	
	1 Biofeedback, Psychology/ (5766)<br>2 Feedback, Sensory/ (546)<br>3 Feedback/ (24530)<br>4 Feedback systems.mp. (359)<br>5 biofeedback.mp. (7227)<br>6 neurofeedback.mp. (291)<br>7 Autogenic Training/ (979)<br>8 or/1-7 (33284)<br>9 limit 8 to (controlled clinical trial or randomized controlled trial) (1734)<br>10 limit 9 to complementary medicine (940)<br><br>Registry:<br>feedback or sensory or 'feedback system*' or biofeedback or neurofeedback or 'bio feedback' or 'autogenic train*'<br><br>Yield = 2109<br><br>*Note: Feedback Systems/ is an Embase term added for good measure.*<br><br>Additional sources consulted:<br>Greenhalgh J, Dickson R, Dundar Y. The effects of biofeedback for the treatment of essential hypertension: a systematic review. Health Technol Assess. 2009 Oct;13(46):1-104.
**Hypnosis**	
	1 Hypnosis/ (7838)<br>2 Hypnosis, Anesthetic/ (508)<br>3 Hypnosis, Dental/ (450)<br>4 hypnotherapy.mp. (801)<br>5 hypnos*.mp. (10239)<br>6 hypnot*.mp. (28442)<br>7 or/1-6 (34762)<br>8 (sedative or hypnosedative or anxiolytic* or Hypnotics).mp. and Sedatives/ (20276)<br>9 7 not 8 (14486)<br>10 limit 9 to (controlled clinical trial or randomized controlled trial) (1027)<br>11 limit 10 to complementary medicine (470)<br><br>Registry:

	hypnos* OR hypnot* OR 'anesthetic hypnosis' OR 'dental hypnosis' OR 'hypnotherapy'<br><br>Yield=780
**Meditation**<br>(includes mindfulness-based therapies)	
	1 Vitalism/ (159)<br>2 vitalism.tw. (133)<br>3 Meditation/ (1122)<br>4 meditat*.tw. (2327)<br>5 mindful$.tw. (1785)<br>6 intentionally-focused awareness.tw. (0)<br>7 (positive adj1 psycholog*).tw. (500)<br>8 (meditative adj2 practice).tw. (18)<br>9 or/1-8 (4768)<br>10 limit 9 to (controlled clinical trial or randomized controlled trial) (402)<br><br>Registry:<br>vitalism OR meditation OR 'meditative practice' OR samu OR 'positive psycholog*' OR 'intentionally focused awareness' OR mindful* OR aware*<br><br>Yield=1259<br><br>Note: We did not include ‘samu’ term in OVID medline because it largely referred to mobile emergency services in France. For CAM purposes, samu refers to meditation while doing chores like cleaning or cooking.
**Relaxation**<br>(includes guided imagery and deep breathing)	
	1 Relaxation/ (1671)<br>2 Rest/ (11087)<br>3 Relaxation Therapy/ (5459)<br>4 (progressive adj2 muscle adj2 relaxation).tw. (289)<br>5 (relax* adj2 technique*).tw. (1207)<br>6 Simonton.tw. (16)<br>7 visuali$ation.tw. (1)<br>8 exp "Imagery (Psychotherapy)"/ (951)<br>9 (guided adj2 imagery).tw. (450)<br>10 sophrology.tw. (88)<br>11 ((post and isometric).tw. and (contracts.ti,ab. or exp contracts/ or contract.tw.)) or hold relax.tw. (27)<br>12 (breathing adj3 exercises).tw. (466)<br>13 (alternate adj3 nostril).tw. (15)<br>14 (abdominal adj2 breath*).tw. (200)<br>15 neidan.tw. (0)<br>16 (unilateral adj2 nostril).tw. (35)<br>17 (forced adj2 nostril).tw. (27)<br>18 deep breathing.tw. (1267)

	19 Muscle Relaxation/ (11163)<br>20 Diastole/ (13795)<br>21 (rebirthing adj3 breath$).tw. (0)<br>22 or/1-21 (46286)<br>23 limit 22 to (controlled clinical trial or randomized controlled trial) (3650)<br>24 limit 23 to complementary medicine (1633)<br><br>Registry:<br>relaxation or rest or relax* or Simonton or visualisation or visualization or imagery or sophrology or 'muscle contract*' or 'hold relax' or 'breathing exercise*' or 'alternate nostril' or 'abdominal breath' or neidan or 'unilateral nostril' or 'forced nostril' or 'deep breathing' or rebirth*<br><br>Yield=3743
**Sensory art therapies**<br>**(includes art, dance, drama, music, and play therapy)**	
	1 Sensory Art Therapies/ (48)<br>2 expressive writing.tw. (101)<br>3 neuroacoustic.tw. (5)<br>4 (humour therapy or humor therapy).tw. (17)<br>5 Laughter Therapy/ (102)<br>6 (therapeutic adj2 humor).tw. (18)<br>7 (therapeutic adj2 humour).tw. (5)<br>8 (colour therapy or color therapy).tw. (14)<br>9 exp color therapy/ (57)<br>10 exp art therapy/ (1008)<br>11 art therapy.tw. (463)<br>12 exp music therapy/ (2039)<br>13 (music adj2 therapy).tw. (1082)<br>14 Dance Therapy/ (172)<br>15 dance therapy.tw. (38)<br>16 Psychodrama/ (993)<br>17 (role adj2 play*).tw. (156051)<br>18 (drama adj3 therapy).tw. (34)<br>19 journaling.tw. (151)<br>20 Bibliotherapy/ (301)<br>21 Animal Assisted Therapy/ (48)<br>22 Equine-Assisted Therapy/ (26)<br>23 Sensory Deprivation/ (4498)<br>24 (sensory adj2 deprivation).tw. (842)<br>25 (restricted adj2 environmental adj2 stimulation).tw. (30)<br>26 Horticultural Therapy/ (9)<br>27 Gardening/ (346)<br>28 hippotherapy.mp. (58)<br>29 trager.mp. (156)<br>30 or/1-29 (166609)<br>31 limit 30 to (controlled clinical trial or randomized controlled trial) (2799)<br>32 limit 31 to complementary medicine (849)<br><br>Registry:

	'sensory art therap*' or art or 'expressive writing' or 'neuroacoustic' or 'humour therapy' or 'humor therapy' or 'laughter therapy' or 'therapeutic humor' or 'therapeutic humour' or 'colour therapy' or 'color therapy' or 'art therapy' or music or dance or psychodrama or drama or writing or reading or journaling or bibliotherapy or 'animal assisted therapy' or 'pet therapy' or equine or horse* or hippotherapy or 'sensory deprivation' or 'horticultural therapy' or gardening or trager<br><br>Yield=1,136
**Tai chi**	
	1 Martial Arts/ (714)<br>2 Tai Ji/ (456)<br>3 tai chi.tw. (606)<br>4 (taiji or tai ji).tw. (43)<br>5 ai chi.tw. (6)<br>6 or/1-5 (1341)<br>7 limit 6 to (controlled clinical trial or randomized controlled trial) (170)<br><br>Registry:<br>'martial arts*' OR 'Tai chi' OR taiji OR 'Tai ji' OR 'Ai Chi'<br><br>Yield=188
**Yoga**	
	1 exp Yoga/ (1220)<br>2 Bhakti.tw. (1)<br>3 Hatha.tw. (70)<br>4 asanas.tw. (53)<br>5 Kundalini.tw. (20)<br>6 bikram.tw. (3)<br>7 yoga.tw. (1336)<br>8 yogi*.mp. (193)<br>9 or/1-8 (1766)<br>10 limit 9 to (controlled clinical trial or randomized controlled trial) (224)<br>11 limit 10 to complementary medicine (223)<br><br>Registry:<br>Yoga OR hatha OR asanas OR kundalini OR bikram OR yogi* OR ashtanga OR raja OR bhakti<br><br>Yield=333
**Therapies using putative energy fields**<br>(distant healing, prayer, qi gong, reiki, spiritual healing, and therapeutic touch)	
	1. Qi/<br>2. Therapeutic Touch/<br>3. Radiesthesia/<br>4. Spiritual Therapies/<br>5. Magic/<br>6. Occultism/

<table>
<tr><td></td><td>7. Witchcraft/
8. Astrology/
9. "Religion and Medicine"/
10. "Religion and Psychology"/
11. Mental Healing/
12. Faith Healing/
13. Spirituality/
14. (distant heal* OR prayer OR qi gong OR qigong OR reiki OR spiritual heal* OR mental heal* OR alternative heal* OR faith heal* OR pranic heal* OR (special AND heal*) OR santeria OR witchcraft OR voodoo OR pray* OR psychospiritual OR therapeutic touch OR healing touch OR caring touch OR healing art* OR huna OR (ch adj i) OR chi kung OR chi gung OR (feng AND shui) OR biofield* OR radiesthesia OR magic OR occultism OR witchcraft OR astrol*).mp.
15. (natural AND healing).ti.
16. (((qi# OR qi) AND healing) OR (pranic AND healing) OR (psychic AND (healing OR surgery))).mp.
17. ((craniosacral OR cranio-sacral) AND (therap* OR treat*)).mp.
18. or/1-17
19. limit 18 to (controlled clinical trial OR randomized controlled trial)
20. limit 19 to complementary medicine

Medline = 4178 RCT or CCT, of which 854 are CAM

Registry:
#45=Qi OR #45='Spiritual Therapies' OR #45='Religion' OR #45=Spirituality OR 'distant heal*' OR prayer OR 'qi gong' OR qigong OR reiki OR 'spiritual heal*' OR 'mental heal*' OR 'alternative heal*' OR 'faith heal*' OR (special AND heal*) OR (pranic AND heal*) OR santeria OR witchcraft OR voodoo OR pray* OR psychospiritual OR 'therapeutic touch' OR 'healing touch' OR 'caring touch' OR 'healing art*' OR huna OR 'ch i' OR 'chi kung' OR 'chi gung' OR (feng AND shui) OR biofield OR radiesthesia OR magic OR occultism OR witchcraft or astrol* OR ((qi* OR qi) AND healing) OR (pranic AND healing) OR (psychic AND (healing OR surgery)) OR ((craniosacral OR cranio-sacral) AND (therap* OR treat*))

Yield = 1210</td></tr>
<tr><td colspan="2">Therapies using veritable energy modalities
(unconventional uses of magnets, phototherapy, electrical stimulation, OR ultrasonic therapy)</td></tr>
<tr><td></td><td>*magnetics/tu OR electromagnetics/tu OR electromagnetic fields/tu OR Magnetic Field Therapy/ OR Transcutaneous Electric Nerve Stimulation/ OR Transcranial Magnetic Stimulation/ OR Electric Stimulation Therapy/

((magnet OR electromagnetic).mp. AND (th.xs. OR therapeutics.ti,ab. OR exp therapeutics/ OR therapy.tw.))
(infrasound.mp. AND (th.xs. OR therapeutics.ti,ab. OR exp therapeutics/ OR therapy.tw.))
Medline

((millimeter wave*).mp AND (th.xs. OR therapeutics.ti,ab. OR exp therapeutics/ OR therapy.tw.))
(mora.tw. AND (th.xs. OR therapeutics.ti,ab. OR exp therapeutics/ OR therapy.tw.))</td></tr>
</table>

	OR ((ultrasound.tw. OR radiofrequenc*.mp.) AND hyperthermia, induced/)<br>((energy AND polarity).mp. AND (wound healing.ti,ab. OR exp wound healing/ OR healing.tw.))<br>(polarity.mp. AND therapy.tw.)<br><br>(transcutaneous electric* nerve stimulation OR transcranial magnetic stimulation OR repetitive transcranial magnetic stimulation OR electrostimulation OR blue light treatment OR (holographic AND repatterning) OR thermochemoradiotherapy OR bioresonance OR bioelectromagnet* OR biomagnets OR ion generat* OR electrodiagnostics OR neural therapy OR neuraltherapy OR pulsed signal therapy OR extracorporeal shockwave therapy OR ESWT OR light therapy OR phototherapy OR heliotherapy OR syntonics OR gravity inversion OR Electroacupuncture OR percutaneous electrical nerve stimulation OR aeroionotherapy OR aerions OR energy polarity healing OR heat therapy OR electrotherap* OR kirlian OR ear candle* OR ear coning OR thermal-auricular OR sonopuncture OR acutonics OR short wave diatherm*).mp.<br><br>MEDLINE 3996 RCT or CCT of which 2284 are in the CAM subset<br><br>Registry<br><br>((magnet OR electromagnetic OR infrasound OR mora OR polarity) AND therap*) OR radiofrequenc* OR 'millimeter wave' OR 'magnetic field therapy' OR magnetics OR electrical OR TENS OR 'transcranial magnetic stimulation' OR 'repetitive transcranial magnetic stimulation' OR electrostimulation OR 'electric stimulation therapy' OR 'blue light treatment' OR (holographic AND repatterning) OR thermochemoradiotherapy OR bioresonance OR bioelectromagnet* OR biomagnets OR 'ion generat*' OR electrodiagnostics OR 'neural therapy' OR neuraltherapy OR 'pulsed signal therapy' OR 'extracorporeal shockwave therapy' OR ESWT OR 'light therapy' OR phototherapy OR heliotherapy OR syntonics OR 'gravity inversion' OR electroacupuncture OR 'percutaneous electrical nerve stimulation' OR aeroionotherapy OR aerions OR 'energy polarity healing' OR 'heat therapy' OR electrotherap* OR kirlian OR 'ear candle*' OR 'ear coning' OR 'thermal-auricular' OR sonopuncture OR acutonics OR 'short wave diatherm*'<br><br>Yield = 2977

General Sources Consulted

1. Martin CW, Advisor SM. Alternative medicine: Some definitions, evidence & references. 2009. Available at: www.worksafebc.com/health_care.../PDF/alternative_medicine.pdf.
2. Loo, May. Integrative Medicine for Children. St. Louis, Mo: Saunders/Elsevier, 2009. (electronic book)
3. National Libary of Medicine, Office of Dietary Supplements. Search Strategy Used to Create the Dietary Supplements Subset on PubMed. 2011. Available at: http://www.nlm.nih.gov/bsd/pubmed_subsets/diet_strategy.html. Accessed March 15, 2012.

4. National Library of Medicine, National Center for Complementary and Alternative Medicine. Search Strategy Used to Create the Complementary Medicine Subset on PubMed. 2012. Available at: http://www.nlm.nih.gov/bsd/pubmed_subsets/comp_med_strategy.html. Accessed March 15, 2012.
5. Bompa T. Theory and methodology of Training : The Key to Athletic Performance. Dubuque [Iowa]: Kendall/Hunt Publishing Co.; 1983.
6. Greenhalgh J, Dickson R, Dundar Y. The effects of biofeedback for the treatment of essential hypertension: a systematic review. Health Technol Assess. 2009 Oct;13(46):1-104.
7. AMED thesaurus
